# Immunotherapeutic Strategies in Cancer and Atherosclerosis—Two Sides of the Same Coin

**DOI:** 10.3389/fcvm.2021.812702

**Published:** 2022-01-13

**Authors:** Felix Sebastian Nettersheim, Felix Simon Ruben Picard, Friedrich Felix Hoyer, Holger Winkels

**Affiliations:** ^1^Department of Cardiology, Faculty of Medicine and University Hospital Cologne, University of Cologne, Cologne, Germany; ^2^Center for Molecular Medicine Cologne (CMMC), University of Cologne, Cologne, Germany

**Keywords:** tumor, atherogenesis, cardiovascular disease, immunotherapy, immunity, T cell, checkpoint inhibition, co-stimulatory molecule

## Abstract

The development and clinical approval of immunotherapies has revolutionized cancer therapy. Although the role of adaptive immunity in atherogenesis is now well-established and several immunomodulatory strategies have proven beneficial in preclinical studies, anti-atherosclerotic immunotherapies available for clinical application are not available. Considering that adaptive immune responses are critically involved in both carcinogenesis and atherogenesis, immunotherapeutic approaches for the treatment of cancer and atherosclerosis may exert undesirable but also desirable side effects on the other condition, respectively. For example, the high antineoplastic efficacy of immune checkpoint inhibitors, which enhance effector immune responses against tumor cells by blocking co-inhibitory molecules, was recently shown to be constrained by substantial proatherogenic properties. In this review, we outline the specific role of immune responses in the development of cancer and atherosclerosis. Furthermore, we delineate how current cancer immunotherapies affect atherogenesis and discuss whether anti-atherosclerotic immunotherapies may similarly have an impact on carcinogenesis.

## Introduction

Although prevention strategies and therapeutic opportunities have been significantly improved during the past decades, atherosclerotic cardiovascular diseases (CVD) and cancer still represent the two most common causes of death worldwide (1). As already recognized by Rudolph Virchow in the nineteenth century (2, 3), the critical role of inflammatory processes in atherogenesis and carcinogenesis is now well-established and has prompted investigation of strategies to combat these deadly diseases by modulating underlying immune responses (4–10). Several anti-cancer immunotherapies, such as cytokines, antibodies targeting immune cell receptors, or immune checkpoints, dendritic cell therapy, and chimeric antigen receptor (CAR) T cell therapy, already found their way into clinical practice and thereby revolutionized cancer treatment (9, 11). In stark contrast, clinically approved immunotherapies for CVD are still not available [except for antibodies targeting proprotein convertase subtilisin/kexin 9 (PCSK9) to lower low-density lipoprotein (LDL) cholesterol, representing an immunotherapeutic approach in a broader sense (12, 13)]. In 2017, the CANTOS trial demonstrated that administration of an antibody directed against the pro-inflammatory cytokine interleukin-1β (IL-1β) reduced cardiovascular events in patients with coronary artery disease (CAD), thereby providing first evidence for effectiveness of an immunotherapy in CVD (14). Yet, this therapy increased the risk of fatal infections and did not reduce mortality, which consequently prevented its approval for treatment of CVD (14). CANTOS illustrated the central dilemma of many immunomodulatory strategies: Broad interventions in the immune system can have detrimental side effects. In general, anti-atherosclerotic strategies are geared toward suppression of vascular inflammation (5, 8, 15), whereas immune-based cancer treatments aim at enhancing immune responses against tumor cells (7, 9). The therapeutic efficacy of several anti-cancer immunotherapies is constrained by their proimmunogenic (and thus proatherogenic) properties, increasing the risk to develop CVD in patients (16). Particularly, immune checkpoint inhibitors directly aggravate atherosclerotic plaque growth in patients (17). Whereas, cancer survival has dramatically improved over the past few decades (18), the exposure of cancer survivors to therapy-induced cardiovascular risk represents an emerging problem, which leads to excess cardiovascular mortality and thus significantly affects long-term prognosis (19–22). This problem is relevant, as the global cancer burden is expected to increase by ~47% within the next 20 years and to reach more than 28 million cases in 2040 (23).

In recent years, vaccination strategies aiming to either induce immune responses against tumor-specific neoantigens (4) or to suppress immunity against atherosclerosis-related autoantigens (6) have emerged. Immunization strategies are promising as they enable specific immunomodulation without impairing host defense responses or accelerating progression of atherosclerosis. Whereas, anti-atherosclerotic vaccination strategies are still in their infancy (6), therapeutic cancer vaccines are already being investigated in clinical trials (4).

In this review, we will provide an overview of current immunomodulatory concepts for treatment of cancer and atherosclerosis with a focus on their reciprocal interactions and consequences. Finally, we will highlight the potential of immunization strategies against cancer and CVD that enable targeted, antigen-specific immunity without affecting the immune system.

## Inflammation and Adaptive Immunity in Atherogenesis

Atherosclerosis involves formation of lipid-laden plaques in large and medium-sized arteries (24), which may rupture or erode and give rise to acute thrombotic vessel occlusion (25). Plaque formation primarily occurs in regions with disturbed blood flow and low endothelial shear stress (26). Such hemodynamic alterations induce a cascade of endothelial dysfunction, subendothelial accumulation and subsequent oxidation of lipoproteins, and finally an inflammatory response that is characterized by monocyte infiltration and foam cell formation (25, 26). Extensive research during the past decades has indicated that plaque-related inflammation is not simply a passive process but is rather orchestrated by an adaptive immune response involving T cells and humoral immunity (27).

**Figure 1:**
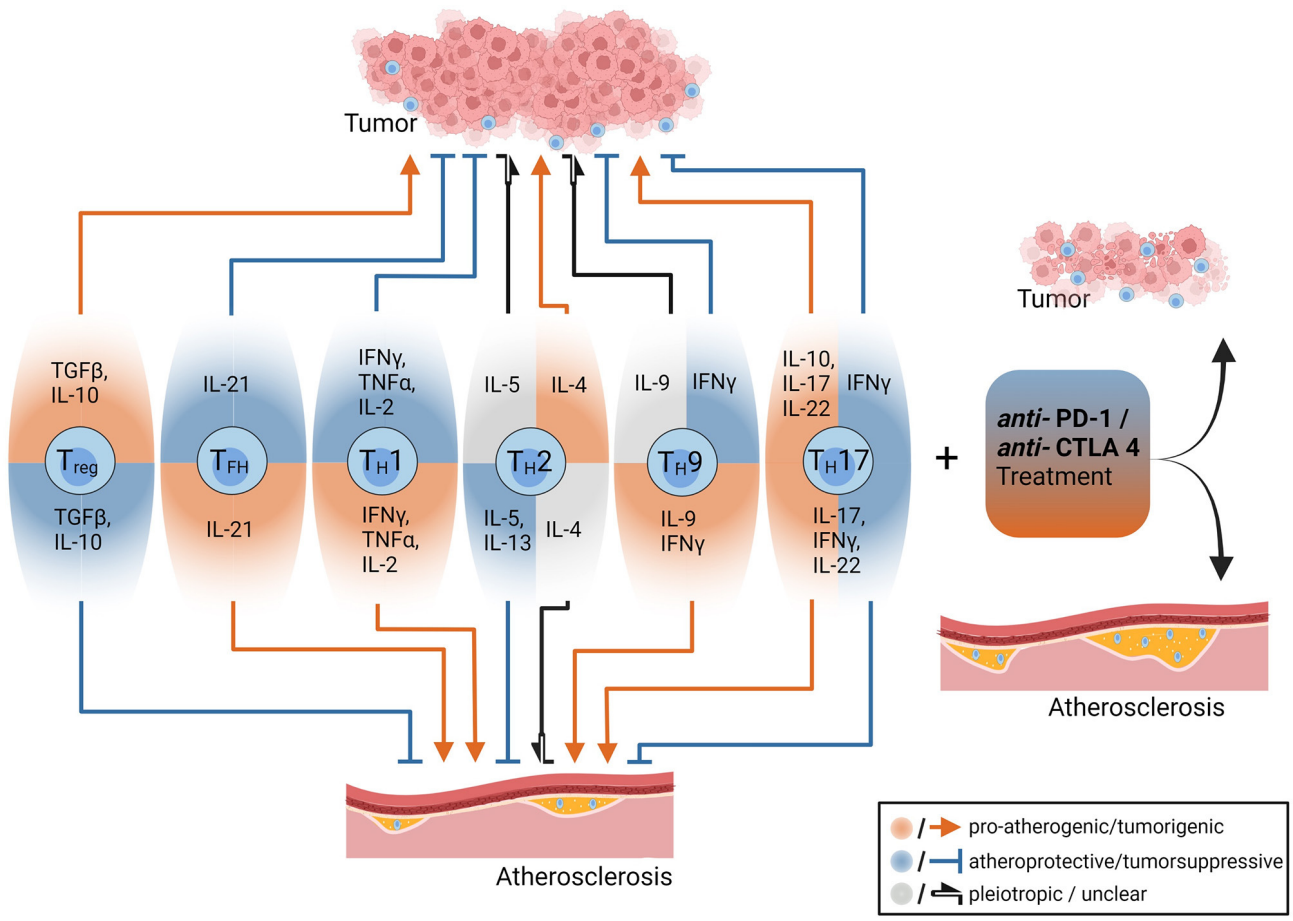
The opposing roles of CD4^+^ T-cells and anti-PD-1/anti CTL4 treatments in the pathogenesis of tumors and atherosclerosis. CD4^+^ T cell subsets and their effector cytokines have different roles in tumors and atherosclerosis. Some cytokines such as interferon gamma have pro-atherogenic (red arrow) and tumor-suppressive effects (blue arrow with blunt end), while Interleukin-10 (IL-10) depending on its cellular source exerts anti-atherogenic and tumor-progressive functions. Treatment with immune checkpoint inhibitors (anti-PD1 and anti-CTLA-4 antibodies) reduces tumor burden but drives atherosclerosis among others by enhancing pro-inflammatory T cell effector functions. The figure was created with Biorender.com.

T cells derive from hematopoietic progenitor cells and undergo a complex maturation and selection process in the thymus, which is characterized by development of a unique, antigen-specific T cell receptor (TCR) through random genetic recombination (28) and elimination of cells that are either non-functional or bind self-antigens with too high-affinity, which are potentially dangerous for the host (29). The high prevalence of autoimmune disorders indicates the insufficiency of this process. Eventually, the TCR and one of its co-receptors CD4 or CD8 are expressed on the T cell surface, which is released into the periphery and circulates through the body to encounter its cognate antigen (30). Activation of a naïve T cell requires two signals. First, the TCR must be bound by its cognate antigen: CD4^+^ T cell activation requires presentation of an antigenic peptide-sequence, the so-called epitope, on major histocompatibility complex class II (MHC-II) molecules, which are exclusively expressed by professional antigen-presenting cells (APCs), such as dendritic cells, macrophages and B cells. CD8^+^ T cells recognize antigens presented on MHC-I molecules, which are expressed by all nucleated cells (31). Second, the T cell must simultaneously receive a proper co-stimulatory signal, that is binding of a specific receptor (such as CD28) by its ligand expressed on the APC (32). Once activated, T cells proliferate and CD8^+^ T cells become cytotoxic, whereas CD4^+^ T cells can differentiate into a variety of different subtypes, which are characterized by expression of specific surface markers, transcription factors (TFs) and cytokines (33). For example, T helper 1 (T_H_1) cells, which are characterized by expression of the TF T-box expressed in T cells (T-bet), exert pro-inflammatory effects through production of interferon gamma (IFN-γ) (Figure 1). In contrast, regulatory T cells (T_regs_), which are characterized by expression of the TF forkhead box protein P3 (FoxP3), produce the anti-inflammatory cytokines IL-10 and transforming growth factor beta (TGF-β) and thus ensure immune tolerance. For a thorough overview of different T cell subtypes and their role in atherosclerosis the interested reader is referred to Saigusa et al. (34).

Presence of T cells in atherosclerotic plaques was firstly described by Hansson and colleagues more than 30 years ago (35). T cells within the plaques were activated (36) and *in vitro* work showed lesional CD4^+^ T cells responding to oxidized low-density lipoprotein (oxLDL), which established the theory of T cells contributing to plaque formation (37). By now, CD4^+^ and CD8^+^ T cell responses against plaque-associated autoantigens have been identified to modulate atherogenesis (34, 38, 39). Whereas, T cell reactivity against LDL was originally thought to be induced by oxidation-dependent generation of neoepitopes representing “altered self” (37), more recent work has identified CD4^+^ T cells responding to peptides of native Apolipoprotein B (ApoB), the core protein of LDL, chylomicrons, and other lipoprotein particles. Several unmodified ApoB-peptides have been found to bind murine and human MHC-II molecules with high affinity and thereby evoke a CD4^+^ T cell response (40–43). Such ApoB-reactive (ApoB^+^) CD4^+^ T cells mainly comprise T_regs_, which confer atheroprotective properties in healthy humans, but coexpress TFs typical of proatherogenic T_H_1 or T_H_17 cells in individuals with subclinical atherosclerosis as determined by carotid ultrasound (42). Preclinical studies further elucidated that CD4^+^ T_regs_, particularly those reactive to ApoB, gradually acquire proatherogenic T_H_1/T_H_17, T_H_1/T_reg_ or T follicular helper (T_FH_) phenotypes during atherogenesis (43–45). Whereas, therapeutic interventions that aim to stabilize and/or expand ApoB^+^ T_regs_ hold promise for atherosclerosis prevention and treatment, immunomodulatory therapies causing destabilization of T_regs_ naturally aggravate progression of atherosclerotic lesions (46).

Besides T cells, humoral immune responses against plaque-associated autoantigens have been implicated in atherogenesis (38, 47). Antibodies directed against oxLDL are detectable in human plaques (48) and in plasma samples of humans with our without atherosclerotic CVD (49, 50). Accordingly, B cells can be found in healthy and atherosclerotic vessels, especially in arterial tertiary lymphoid organs located in the adventitia (51). Autoantibodies against oxLDL were shown to block uptake of oxLDL by macrophages (52, 53) and to confer atheroprotection (54). Genetic B-cell depletion aggravated atherosclerosis in LDL-receptor-deficient (*Ldlr*^−/−^) mice (55). Yet, depletion of mature B cells through administration of a CD20 monoclonal antibody was unexpectedly atheroprotective in Apolipoprotein E-deficient (*Apoe*^−/−^) and *Ldlr*^−/−^ mice (56, 57). This treatment preserved the production of natural IgM antibodies directed against oxLDL but reduced anti-oxLDL IgG antibodies (56). Adoptive transfer of B2 B cells, but not B1 B cells, to lymphocyte or B cell deficient *Apoe*^−/−^ mice was proatherogenic (57). B cells mainly consist of B2 B cells (which are thus termed conventional B cells) that undergo maturation in the spleen and can produce high-affinity IgG antibodies after receiving T cell help (58). B1 B cells represent a specialized B cell subpopulation: They develop in the fetal and neonatal period, harbor the capability of self-renewal, mainly reside in body cavities and are characterized by the production of so called “natural” IgM antibodies (59). Taken together, different B cell subsets and antibody subtypes may exert diametral functions in atherogenesis and therapeutic modulation of humoral immune responses could represent an attractive anti-atherosclerotic strategy but also promote atherosclerosis progression.

## Inflammation and Adaptive Immunity in Cancer

In the nineteenth century, the German physicians Rudolph Virchow, Wilhelm Busch and Friedrich Fehleisen independently hypothesized that inflammation may affect carcinogenesis (2, 60). Whereas, Virchow assumed that leukocyte infiltrates represented an underlying cause of cancer (2), Busch and Fehleisen suggested that inflammation may reverse tumorigenesis (60). After independently observing involution of malignancies in patients with erysipelas, they demonstrated tumor regression in cancer patients upon intentional infection with bacteria isolated from erysipelas (61, 62). Later, the American surgeon William Coley reported disappearance of tumors in patients with inoperable sarcoma or other types of cancer after treatment with heat-inactivated bacteria which was termed “Coley's Toxin” (63). In 1909, Paul Ehrlich suggested that cellular immunity may recognize neoplastic cells and protect from tumor development, although he was not able to experimentally substantiate this hypothesis (64). First experimental proof for anti-tumor immune responses was provided by Gross and Foley around 1950 (65, 66) and Paul Ehrlich's concept was adopted by Lewis Thomas and Sir Frank Macfarlane Burnet who proposed that lymphocytes recognize and target cancer cells through their expression of tumor-specific antigens, similar to homograft rejection (known as immunosurveillance) (7, 67). Yet, this theory was abandoned after immunologically impaired animals, such as athymic nude mice, showed similar susceptibility to experimentally induced tumors (68, 69). Several limitations of these experiments became evident: Nude mice—despite lack of T and B cells—are not completely immunocompromised and especially susceptible to 3-methylcholanthrene, the chemical carcinogen which was used for tumor induction (due to expression of highly active enzyme isoforms involved in biotransformation of the chemical) (68). Novel immunocompromised mouse models with pure genetic backgrounds demonstrated that lymphocyte deficiency (70), lack of perforin (an important component of cytotoxic T lymphocyte granules) (71, 72), and ablation of proinflammatory cytokine signaling (70–73) increased tumor susceptibility in mice, which led to the renaissance of the immunosurveillance theory (68). This preclinical evidence was supported by studies reporting an increased cancer risk in immunocompromised patients (74–76) and that tumor lymphocyte infiltration predicts better outcome (77, 78). Despite overall proof in support of the immunosurveillance theory was provided, subsequent work demonstrated that immunity may also exert tumor-sculpting effects (68): Tumors derived from immunocompromised mice were rejected more frequently when transplanted into immunocompetent recipients than tumors derived from wild-type controls (70, 72, 79, 80). Thus, the immune system of the wild-type donors must have shaped tumors to become less immunogenic and more resistant to the hosts (uncompromised) immune response.

To account for the dual role of the immune system in tumor development, G.P Dunn and R.D. Schreiber proposed the groundbreaking “immunoediting” or “three E's” theory in 2002 (68), which is the current explanation of tumor-related immune responses (81). The theory involves three processes: (1) In the elimination phase, which conforms to the original immunosurveillance theory, tumor cells are targeted by innate and, subsequently, adaptive immune cells including tumor antigen-specific CD4^+^ and CD8^+^ T cells. If the immune system is successful in destroying all tumor cells, progression to subsequent phases is prevented. (2) In the equilibrium phase, that may last for years, tumor cells that have survived the initial elimination process and the ongoing immune response are in balance. Although tumor growth is still under immunological control, the immune system fails in eliminating all tumor cells, and thus causes selection pressure on the surviving variants. (3) In the escape phase, tumor cells that have undergone extensive immunoediting, evade immunological control, and expand rapidly, resulting in development of clinically apparent disease (68).

More recently, immunological processes underlying the three phases of immunoediting have been characterized in greater detail, which has led to development of immunotherapies that efficiently enhance anti-tumor immune responses (82, 83). Modern technologies have enabled identification of tumor-specific, MHC-I and -II restricted neoantigens and detection of CD8^+^ and CD4^+^ T cells responding to such neoantigens (82, 83). These technologies include deep-sequencing approaches to determine the “mutanome,” that is the entirety of tumor-specific mutations, followed by *in-silico* prediction algorithms to identify mutation-specific epitopes capable of binding to MHC-I or -II molecules (84–88). In a second step, immunogenicity of identified epitopes is verified through T cell restimulation assays of peripheral blood mononuclear cells from the sequenced patient (84, 86–89) or MHC-I (85, 90) and -II (91, 92) tetramers or multimers detecting tumor-neoantigen-specific CD8^+^ and CD4^+^ T cells, respectively. Finally, multimer-selected neoepitope-specific CD8^+^ and CD4^+^ T cells can be phenotyped by flow-cytometry or (single-cell) RNA-sequencing approaches (90–92). Whereas, initial studies were focused on the role of CD8^+^ T cells in mediating anti-tumor immunity (85, 87, 93), subsequent work established that the immunogenic mutanome—against former expectations- predominantly induced a CD4^+^ T cell response in mice and humans (86, 89–91, 94). Tumor neoepitope-reactive CD4^+^ T cells were crucially involved in generation of potent anti-tumor CD8^+^ T cell responses (92, 95). This T cell help is mainly mediated by interactions of CD40 ligand (CD40L), which is expressed on the surface of activated CD4^+^ T cells, and CD40 on the surface of APCs (96). Additionally, CD4^+^ T cells may exert direct anti-neoplastic activity through production of pro-inflammatory cytokines or execution of cytotoxic signals on tumor cells and aid in B cell mediated humoral anti-tumor responses through CD40L signaling (97).

Neoepitope-specific CD4^+^ T cells of the T_H_1 subtype are involved in anti-tumor responses (92). Adoptive transfer of neoepitope-specific CD4^+^ T_H_1 cells led to tumor regression in a patient with metastatic cholangiocarcinoma (98). In line with this, high levels of circulating tumor-antigen-specific T_H_1 CD4^+^ T cells and low levels of CD4^+^ cells co-expressing the immune-checkpoints programmed cell death protein 1 (PD-1) and T cell immunoglobulin and mucin-domain containing-3 (Tim-3) predict better survival in lung cancer patients (99). In contrast, high levels of tumor-infiltrating T_regs_, which can be found in various cancer types, are associated with poor prognosis (100, 101). Animal studies identified tumor-induced conversion of CD4^+^ non-T_regs_ into T_regs_ as an important mechanism of immune escape (102) and, accordingly, circulating tumor-antigen-specific T_regs_ can be detected in cancer patients but not in healthy individuals (103). Other T helper cell subsets, such as T_H_2 and T_H_17 cells, can also be found in the tumor microenvironment, but their specific role in tumor immunity and prognostic importance are still under debate (104, 105).

In conclusion, CD4^+^ T cells responding to tumor-specific neoepitopes play an important role in mediating anti-tumor immune responses. Yet, tumor cells may engage various escape mechanisms to acquire resistance to this response, which include induction of CD4^+^ T cell phenotype switching from proinflammatory anti-neoplastic T_H_1 cells into immunosuppressive and thus tumor growth-promoting T_regs_ (105).

## Effects of Clinically Approved Cancer Immunotherapies on Atherogenesis

Several immunotherapeutic strategies aim at preserving or restoring anti-tumor immune responses. Yet, the opposing roles of adaptive immunity in atherosclerosis and cancer development (Figure 1) implicate that such therapeutic approaches might involve proatherogenic side effects (17), especially if they are not antigen-specific but affect the immune system as a whole. In contrast, B cell depleting antibodies and antibodies targeting growth factor receptors overexpressed by tumor cells may confer atheroprotection. In the following section we will discuss effects of clinically approved cancer immunotherapies on atherogenesis and delineate their mechanistic background (an overview of these effects is given in Table 1).

**Table 1:** Pro-atherogenic and athero-protective effects of current cancer immunotherapies.

**Type of immunotherapy**	**Specific approach/substance**	**Compounds**	**Effect on atherosclerosis in clinical trials**	**Effect on atherosclerosis in animal studies**	**Potential mechanisms**
Immune checkpoint inhibitors	Anti-CTLA4-Abs	Ipilimumab	↑ (17, 106)	↑ (107–111)	-Increased plaque-infiltration by CD4^+^ and CD8^+^ T cells -Higher expression of proinflammatory cytokines (IFN-γ and TNF-α) by T cells -Enhanced T cell activation
	Anti-PD1-Abs	Pembrolizumab, Nivolumab, Cemiplimab, Dostarlimab			
	Anti-PD-L1-Abs	Atezolizumab, Durvalumab, Avelumab			
Monoclonal antibodies	Anti-CD20-Abs	Rituximab, Obinutuzumab, Ofatumumab	↓ (112–114)	↓ (56, 57)	-Depletion of mature B cells and reduction of anti-oxLDL IgG antibodies
	VEGF inhibitors	Bevacizumab, Ramucirumab	↑ (115–117)	↑ (118)	-Induction of an inflammatory endothelial cell phenotype and impairment of endothelial function -Reduction and functional impairment of T_regs_ and induction of proinflammatory T_H_1 cells
	EGFR targeting Abs	Cetuximab, Necitumumab, Panitumumab	–	↓ (119–121)	-Reduced accumulation of macrophages in plaques -Reduced lipid uptake by macrophages and reduced foam cell formation -Reduced CD4^+^ T cell activation, proliferation and plaque infiltration -Reduced pro-inflammatory cytokine production Reduced SMC proliferation
Cytokines	IFN-α	Interferon alfa	↑ (122, 123)*	↑ (124)	-Increased plasma cholesterol and triglyceride levels -Induction of lipid uptake by macrophages and increased foam cell formation -Inhibition of T_reg_ activation and proliferation -Direct stimulation of cytotoxic CD4^+^ T cell function -Sensitization of antigen-presenting cells toward pathogen-derived TLR4 ligands
	IL-2	Aldesleukin	High-dose: ↑ (125) Low-dose: ?**	High-dose: ↑ (126) Low-dose***: ↓ (127)	-High-dose: unspecific expansion of T cells- Low-dose: selective expansion of functional T_regs_
Antifolate	DHFR inhibition	Methotrexate	- (128)	↓ (129)****	-Attenuation of monocyte maturation and recruitment -Modulation of lipoprotein transcellular transport -Reduction of pro-inflammatory cytokine production

↑ = proatherogenic effect. ↓ = atheroprotective effect. *These studies demonstrated an association between plasma IFN-α levels and atherosclerosis. A direct effect of IFN-α administration on atherosclerosis has not yet been shown in clinical trials. **Currently investigated in the “Low-dose interleukin-2 in patients with stable ischemic heart disease and acute coronary syndromes (LILACS)” trial. ***and complexed with a specific anti-IL2-mAB (JES6-1A12).****delivered via nanoparticles.

*Abs, monoclonal antibodies; CTLA4, cytotoxic T lymphocyte antigen 4; DHFR, dihydrofolate reductase; EGFR, epidermal growth factor receptor; IFN-α, interferon alpha; IFN-γ, interferon gamma; IL2, interleukin 2; mAB, monoclonal antibody; oxLDL, oxidized low density lipoprotein; PD-1, programmed cell death protein 1; PD-L1, programmed cell death ligand 1; SMC, smooth muscle cell; TLR4, toll-like-receptor 4; TNF-α, tumor necrosis factor alpha; VEGF, vascular endothelial growth factor*.

### Immune Checkpoint Inhibitors (ICIs)

Immune checkpoints refer to a variety of regulatory pathways that exert inhibitory actions on adaptive immune cells and beyond and are thus critical for preservation self-tolerance and prevention of exaggerated immune responses (130). The Nobel prize winning discoveries of James P. Allison and Tasuku Honjo, who unraveled that tumor cells may engage immune-checkpoint pathways to escape from anti-tumor immune responses, have paved the way for the development of monoclonal antibodies against these molecules—immune checkpoint inhibitors (ICIs) (131, 132). Ipilimumab inhibits the cytotoxic T lymphocyte antigen 4 (CTLA-4) and was shown to improve overall survival in patients with metastatic melanoma (133), which made it the first ICI approved by the Food and Drug Administration (FDA) in 2011 (134). Subsequently, four antibodies (pembrolizumab, nivolumab, cemiplimab, and dostarlimab) targeting the co-inhibitory programmed cell death protein 1 (PD-1) and three antibodies (atezolizumab, durvalumab, and avelumab) directed against the programmed cell death ligand 1 (PD-L1) were demonstrated to effectively improve survival in several malignancies (134, 135) which led to the FDA-approval for treatment of 19 different cancer types and two tissue-agnostic conditions [that is a tumor with a specific genetic alteration regardless of the cancer type and location (136)]. ICIs have become a cornerstone of modern cancer therapy and nowadays more than 40% of cancer patients are eligible for ICI treatment (137).

Given that immune checkpoints represent important regulators of physiological immune responses, ICI therapy can naturally involve inflammatory side effects, which are referred to as immune-related adverse events (IRAEs) (138). Although the precise pathomechanisms of such IRAEs are not yet fully clear, unconstrained activation of autoreactive T cells is suggested to play a dominant role (138). CTLA-4 and PD-1 are co-inhibitory molecules expressed on the cell surface of CD4^+^ and CD8^+^ T cells (139, 140). When bound by their ligands—CD80/CD86 and PD-L1/PD-L2—CTLA-4- and PD-1 suppress activation of T cells (140). As mentioned above, T cell activation requires simultaneous engagement of the TCR by its cognate antigen and proper costimulatory signals (32). Activation of CD28, the prototype co-stimulatory molecule, by its ligands CD80 or CD86 induces high T cell surface expression of the co-inhibitory molecule CTLA-4 (141). CTLA-4 binds CD80/CD86 with much higher affinity than CD28. However, in contrast to CD28, CTLA-4 does not exert stimulatory but inhibitory signals and thus attenuates T cell activation (141). Given that CD80/CD86 are expressed on the surface of APCs, CTLA-4 inhibits T cell activation mainly in the priming phase. Prolonged TCR stimulation during an ongoing immune response induces PD-1 expression on the cell surface of T cells (141). When bound by its ligands PD-L1 or PD-L2, which can be expressed by tumor cells, PD-1 attenuates TCR-signaling and thus reduces T cell proliferation and cytokine production. Thus, PD-1 mediates T cell inhibition in the effector phase and is used as a marker of T cell exhaustion (141). Consequently, antibody-mediated inhibition of CTLA-4, PD-1 and PD-L1 enhances T cell activation. IRAEs can affect almost every organ and mostly occur within 2–16 weeks after treatment initiation (138, 142). According to a recent meta-analysis including 36 phase II and III randomized controlled trials (RCTs), the pooled incidence of all IRAEs ranges between 54 and 76% (143). Whereas, the incidences of specific IRAEs depend on the ICI used and several other factors, the integumentary, gastrointestinal, endocrine, hepatic, and pulmonary systems are overall most commonly affected (143, 144). In a meta-analysis of 112 trials including 19,217 patients, IRAE-associated fatality rates ranged between 0.36% for anti-PD-1 mono-therapy and 1.23% for PD1/PD-L1 plus CTLA-4 combinational therapy and were most commonly caused by colitis, pneumonitis, hepatitis, myocarditis and neurotoxic effects (145). Cardiovascular IRAEs, which include myocarditis, pericardial diseases, heart failure, dyslipidemia, myocardial infarction, and cerebral arterial ischemia, are, overall, relatively rare with an incidence ranging between ~3 and 20 per 1,000 patients (146). Yet, cardiovascular toxicities are severe in over 80% of cases (147) and myocarditis, which carries the highest fatality risk of all IRAEs (40–50%), is of particular prognostic relevance (145, 147).

Besides acutely occurring cardiovascular IRAEs, recent evidence has suggested that ICI therapy may promote atherogenesis (148, 149). In a retrospective analysis of 1,215 patients treated with ICIs, atherosclerotic cardiovascular events (CVE) occurred in 1% within a follow-up period of 6 months (150). In three meta-analyses, the ICI-related incidence of myocardial infarction and stroke ranged from 0.4 to 1.0% and 1.1 to 2.0%, respectively (149). Yet, the majority of studies included in these meta-analyses were not specifically designed to assess CVE and may thus underestimate incidences (149). To evaluate the ICI-related risk of atherosclerotic CVE (defined as the composite of myocardial infarction, coronary revascularization, and ischemic stroke), Drobni et al. analyzed event-rates in 2,842 patients treated with ICIs and matched controls (17). Additionally, a case-crossover analysis was performed, in which event rates within the 2 years before (control period) and the 2 years after (at-risk period) initiation of ICI therapy were compared. ICI therapy was associated with a 3-fold and almost 5-fold higher risk of atherosclerotic CVE in the matched-control study and case-crossover analysis, respectively (17). In a nested imaging substudy including 40 patients, a 3-fold increase in aortic atherosclerotic plaque volume progression from 2.1%/year before to 6.7%/year after ICI initiation could be detected (17). Another recent study retrospectively analyzed 2-[^18^F]fluorodeoxyglucose (FDG) positron emission tomography/computed tomography scans, which had been performed in 20 melanoma patients before and during ICI treatment (mean time interval: 4.4 months) (106). A significantly increased FDG uptake in large arteries after ICI treatment initiation could be detected, pointing toward an ICI-related induction of arterial inflammation (106). In accordance to these clinical findings, a series of animal studies reported enhanced plaque inflammation and accelerated atherogenesis in *LDLr*^−/−^ mice genetically deficient for or treated with inhibitory antibodies against PD-1, PD-L1 and CTLA-4 (148, 149). This was accompanied by an increased number of plaque-infiltrating CD4^+^ and CD8^+^ T cells (107–111), higher expression of proinflammatory cytokines [IFN-γ and tumor necrosis factor alpha (TNF-α)] by T cells (107, 111), and enhanced T cell activation (108, 110, 111).

Collectively these data emphasize that ICI therapy promotes atherogenesis and substantially increases the risk of atherosclerotic CVE. Presumably, ICIs exert their proatherogenic effects—at least in part—through disinhibition of T cells responding to plaque-associated autoantigens. Atherosclerosis is a slowly progressing disease and all above-mentioned clinical studies were limited by relatively short follow-up periods. As indications for ICI therapy are rapidly expanding and cancer-survival has dramatically improved in recent years, the detrimental impact of ICIs on atherogenesis will, therefore, likely become a more relevant health issue in the future.

### Antibody Therapy

Since the FDA approval of muromonab-CD3, a monoclonal antibody targeting the T cell co-receptor CD3, for the prevention of transplant rejection in 1986, more than 100 therapeutic antibodies have been included in clinical practice (135). Rituximab, a monoclonal antibody targeting the B cell receptor CD20, was approved for treatment of follicular lymphoma in 1997, which opened the door for the use of antibodies in cancer therapy (151). Cancer has emerged as the most common condition for antibody therapy with currently over 40 FDA-approved antibodies (including ICIs) for treatment of several cancer types (135). Antibodies can target cancer through several mechanisms, including direct tumor cell killing, immune-mediated tumor cell-killing, and inhibition of neovascularization or stroma cells (152, 153). Direct tumor cell killing can be achieved through eliciting agonistic activity to apoptosis-promoting receptors, inhibiting growth factor receptor signaling, neutralizing key enzymes, or delivering cytotoxic agents into the cell (152, 153). Mechanisms of immune-mediated tumor cell killing include induction of phagocytosis, complement-activation and cellular toxicity (152, 153). Besides ICIs, the use of several other monoclonal antibodies in cancer therapy is constrained by their cardiovascular side effects, which include myocarditis, heart failure, arrhythmia, orthostatic dysregulation and atherosclerotic cardiovascular events (16). Fortunately, the latter complication is rare and some antibodies can even confer atheroprotective effects.

#### Antibody-Mediated B Cell Depletion

During the past two decades, B cell depleting strategies have been used for treatment of B cell lymphoma and several autoimmune diseases, including rheumatoid arthritis, systemic lupus erythematosus (SLE) and multiple sclerosis (MS) (151, 154). In addition to rituximab and other antibodies targeting CD20 (e.g., obinutuzumab and ofatumumab), antibodies directed against the B cell surface proteins CD19 (blinatumomab), CD22 (inotuzumab ozogamicin and moxetumomab pasudotox), CD38 (daratumumab and isatuximab) and CD319 (elotuzumab) have been approved to treat these conditions (153).

As mentioned above, depletion of B cells through administration of a CD20-specific antibody ameliorated atherogenesis in *Apoe*^−/−^ and *Ldlr*^−/−^ mice (56, 57). In line with this, treatment of *Apoe*^−/−^ mice with a monoclonal antibody targeting B cell activating factor-receptor (BAFFR) to selectively deplete mature B2 cells while sparing B1 cells conferred atheroprotection (155). Similarly, antibody-mediated inhibition of the cytokine B cell–activating factor (BAFF) reduced atherosclerosis in *Apoe*^−/−^ and *Ldlr*^−/−^ mice (156). These findings were recently confirmed in a clinical study: Patients who received rituximab therapy after kidney transplantation had a significantly lower rate of atherosclerotic CVE during 8 years of follow-up as compared to propensity-matched controls (112). Accordingly, rituximab therapy was shown to reduce carotid intima media thickness (113) and to improve flow mediated dilation of the brachial artery, a non-invasive marker of endothelial function (114). The effect of other B-cell–depleting antibodies (including those targeting receptors predominantly expressed on antibody-secreting plasma cells, such as CD39 and CD319) on atherosclerosis has not yet been investigated and the role of plasma cells in atherogenesis is not yet clear (157). Depletion of IgG-producing plasma cells reduced atherosclerotic plaque development in *Apoe*^−/−^ and *Ldlr*^−/−^ mice (158, 159) but associated with plaque instability, which may have deleterious consequences in patients with preexisting atherosclerosis (158). Evidence from preclinical and clinical studies indicate that antibodies targeting CD20 may confer atheroprotection, but these early findings will have to be confirmed in larger clinical trials.

#### Antibodies Targeting Vascular Endothelial Growth Factor

Bevacizumab was the first clinically approved monoclonal antibody targeting vascular endothelial growth factor (VEGF) (160). Originally thought to exert antineoplastic actions exclusively via inhibition of tumor angiogenesis, VEGF-targeted therapies have been demonstrated to arrest tumor growth through a variety of mechanisms, which are not yet fully understood (160). In addition to bevacizumab, an antibody targeting VEGF receptor 2 (ramucirumab) and small-molecules inhibiting VEGF receptor tyrosine kinases (sorafenib and sunitinib) have been FDA-approved (153). A major drawback of VEGF inhibitors is their tendency to induce atherosclerotic CVE. Recent meta-analyses including up to 22 studies reported a ≈ 1.4- to 2.5-fold higher risk of arterial ischemia in patients treated with bevacizumab (115–117). High-dose bevacizumab therapy was even associated with a 4.4- and 6.7-fold higher risk of cardiac and cerebral ischemia, respectively (115). A preclinical study confirmed and mechanistically substantiated these findings by demonstrating that administration of a VEGF-targeting antibody impaired endothelial function and increased atherosclerotic lesions by 33% in *Apoe*^−/−^ mice (118). Accordingly, VEGF inhibitors were shown to induce an inflammatory phenotype in cultured human coronary artery endothelial cells (161). Besides affecting endothelial function, VEGF inhibitors may decrease the number of T_regs_ and impair their suppressive capacity, reduce expression of co-inhibitory T cell molecules, and thus induce proinflammatory T_H_1 responses (162). Although experimental proof is missing, these immunological effects might contribute to the proatherogenic properties of VEGF-inhibiting antibodies (162).

#### Antibodies Directed Against Epidermal Growth Factor Receptors

Receptors of the epidermal growth factor receptor family, such as epidermal growth factor receptor (EGFR) or human epidermal growth factor receptor 2 (HER2/neu), may be overexpressed by tumor cells of several cancer types which can thus acquire the capability of autonomous and uncontrolled proliferation (163, 164). Overexpression of EGFR or HER2/neu is a strong predictor of a negative prognosis in a variety of malignancies (165, 166) and the development of monoclonal antibodies targeting such receptors has advanced cancer treatment. Early clinical studies and large-scale phase 3 trials showed improved outcome in patients with metastatic breast cancer and gastric cancer treated with trastuzumab (targeting HER2/neu) and patients with metastatic colorectal cancer and head and neck cancer treated with cetuximab (directed against EGFR) (167–170). Further HER2/neu and EGFR targeting antibodies have been clinically approved (153). A major drawback of growth factor receptor targeting antibodies (especially trastuzumab) is their potential to induce heart failure, which occurs in up to 20% of all cases (167, 171) and is 1.7 to 4 times more frequently compared to standard chemotherapy (172–174). Accordingly, mice lacking Her2/neu were demonstrated to develop dilated cardiomyopathy (175).

Direct effects of antibodies targeting growth factor receptors on atherogenesis have not yet been reported in clinical trials. Nevertheless, EGFR was detected in human atherosclerotic plaques (176) and increased HER2/neu plasma levels were shown to be associated with a higher risk of CAD (177). In line with this, evidence from preclinical studies indicated that inhibition of growth factor signaling may confer atheroprotection (119–121). In two elegant studies, Zeboudj, Ait-Oufella and colleagues demonstrated that cell-specific depletion of EGFR either in myeloid cells (119) or in CD4^+^ T cells (120) protected *Ldlr*^−/−^ mice from atherosclerosis. EGFR deficiency in myeloid cells limited macrophage accumulation within plaques and lipid uptake by macrophages, whereas CD4^+^ T cell-specific depletion of EGFR reduced CD4^+^ T cell activation, proliferation and infiltration in atherosclerotic lesions. Both cell-specific EGFR deletions were accompanied by reduced pro-inflammatory cytokine production (119, 120). Despite these promising findings, some uncertainties regarding the mechanistic implication of EGFR and its ligands in atherogenesis remain (178), beyond EGFR's profound immunomodulatory role systems-wide must be taken into account (179). Whether atheroprotective effects of growth factor receptor targeting antibodies also apply to humans is still unclear.

### Cytokine Therapy

A variety of cytokines may exert significant anti-neoplastic effects either by directly inhibiting proliferation and inducing apoptosis of tumor cells or by stimulating anti-tumor immune responses (180, 181). Despite promising findings in early preclinical studies, utilization of cytokines as cancer therapeutics was later demonstrated to involve several limitations which hindered broad translation of this treatment approach into clinical practice (180, 181). Nevertheless, IFN-α and IL-2 were clinically approved for the treatment of different malignancies such as hairy cell leukemia, follicular non-Hodgkin lymphoma, melanoma, and Kaposi's sarcoma (IFN-α) or renal cell carcinoma and melanoma (IL-2) (180, 181).

#### Interferon Alpha (IFN-α)

Clinical application of IFN-α is particularly limited by its proatherogenic properties (182, 183). *Ldlr*^−/−^ mice treated with IFN-α had accelerated atherosclerosis and increased plasma cholesterol and triglyceride levels (124). Several other proatherogenic effects of IFN-α have been reported, such as induction of lipid uptake by macrophages and foam cell formation (184, 185), inhibition of T_reg_ activation and proliferation (186, 187), direct stimulation of cytotoxic CD4^+^ T cell function (188), and sensitization of antigen-presenting cells toward pathogen-derived toll-like receptor 4 (TLR4) ligands (189). Clinical studies demonstrated that plasma type I IFN (IFN-α and -β) levels are associated with atherosclerosis development in patients with SLE (122) and human immunodeficiency virus-1 (HIV-1) infection (123). Experimental evidence suggested that IFN-α directly promotes atherogenesis by impairing vascular repair (190, 191) or inducing endothelial dysfunction (192) and may thus causally contribute to the highly increased risk of atherosclerotic CVE in SLE patients, which is not adequately explained by traditional risk factors (193). For a thorough review on the impact of IFN-α on different atherosclerosis-associated cell types and clinical implications the interested reader is referred to Chen et al. (182).

#### Interleukin 2 (IL-2)

IL-2 was originally termed T cell growth factor as it was first identified as a component of T cell culture fluids that induced proliferation of antigen-activated T cells (194, 195). It was thought to act as a crucial mediator in T cell immune responses and to play an important role in host response and tumor control, which led to test high-dose IL-2 as a novel cancer treatment in the mid 1980s (196). Although limited by toxicities such as capillary leak syndrome, fever, chills, malaise and arthralgias, this approach facilitated significant tumor regression and emerged as the first effective immunotherapy for human cancer (196). Yet, IL-2 deficient mice developed severe lymphoproliferation and autoimmunity which pointed toward an additional important role of the cytokine in maintaining self-tolerance (197, 198). Subsequent studies revealed that T_reg_ generation is dependent on IL-2 (195). T_regs_ express increased levels of the high-affinity IL-2 receptor alpha chain (IL-2Rα, also known as CD25) compared to effector T cells (T_eff_ cells) and are thus more sensitive for IL-2 (199). Accordingly, daily low-dose IL-2 therapy stimulated selective expansion of functional T_regs_ through increased proliferation, thymic export and resistance to apoptosis (while only minimally affecting conventional CD4^+^ T cells) and thus led to a substantial clinical improvement in patients with active chronic graft-vs.-host disease (200, 201).

The specific role of IL-2 in atherogenesis has not yet been fully clarified. Increased IL-2 serum levels were shown to be associated with carotid artery intima-media thickness (202), a sonographic marker of atherosclerosis, and CAD (203). An early clinical study reported atherosclerotic CVE in 3.8% (angina or ischemic changes in 2.6% and myocardial infarction in 1.2%) of patients who received IL-2 for cancer therapy (125). Accordingly, IL-2 administration (2 ×10^4^ units twice weekly for a period of 6 weeks) accelerated atherogenesis in *Apoe*^−/−^ mice, whereas administration of an antibody targeting IL-2 was atheroprotective (126). Treatment of *Ldlr*^−/−^ (204) and *Apoe*^−/−^ mice (127) with low-dose IL-2 complexed with a specific IL-2 monoclonal antibody (JES6-1A12) conferred significant atheroprotection by inducing a substantial expansion of T_regs_ in atherosclerotic lesions and several other tissues. Neither IL-2 nor the anti-IL2 antibody alone affected atherogenesis (127) and the observed anti-atherogenic efficacy depended on the antibody clone used. Administration of IL-2 complexed with another IL-2 antibody clone (SAB6) induced expansion of natural killer (NK) and CD8^+^ T cells (205). A subsequent study unraveled the mechanism by which the two different antibody complexes selectively induce expansion of T_regs_ or T_eff_ cells: (1) JES6-1 sterically blocks the interaction of IL-2 with IL-2Rβ and IL-2Rγ and allosterically disrupts binding of IL2 to IL-2Rα, thereby favoring activation of T_regs_ with high IL-2Rα expression; (2) S4B6 sterically hinders IL-2/IL-2Rα interaction and enhances IL-2/IL-2Rβ interaction, thus stimulating all IL-2-responsive T cells (206). More recently, a human anti-IL2-antibody (F5111.2) was developed that selectively promotes T_reg_ expansion when complexed with human IL-2 by inducing similar conformational changes (207). Administration of IL2-F5111.2 complexes yielded substantial therapeutic efficacy in humanized animal models of different autoimmune diseases, such as type 1 diabetes, autoimmune encephalomyelitis or xenogeneic graft-vs.-host disease (207). Whether such approach might be translatable into clinical practice has not yet been determined. The randomized, double-blind, placebo-controlled LILACS trial (NCT03113773) examined whether solely administering low-dose IL-2 is safe and effective in patients with stable ischemic heart disease and acute coronary syndrome (208). The study has been completed and its results are awaited for publication.

In conclusion, current evidence suggests that high-dose IL-2 therapy promotes atherogenesis through induction of pro-inflammatory T_eff_ cell responses, whereas administration of low-dose IL-2 might confer atheroprotection by selectively stimulating expansion of T_regs_. Complexing IL-2 with specific anti-IL-2 antibodies might even enhance the latter effect through augmenting the selectivity to IL-2Rα, which is highly expressed on T_regs_.

### Methotrexate

Methotrexate (MTX) is a structural analog of folic acid (Vitamin B9) that inhibits enzymes involved in folate metabolism, such as dihydrofolate reductase (DHFR), and thus limits cellular division (209). DHFR catalyzes conversion of dihydrofolate to tetrahydrofolate, which acts as an important coenzyme in synthesis of pyrimidine and purine. In 1948 Farber et al. firstly reported that treatment with the folate analog aminopterin enabled temporary remission in childhood leukemia (210). Subsequently, MTX (initially termed amethopterin) was found to have better pharmacological properties than aminopterin (211) and thus emerged as one of the most extensively used chemotherapy agents for a variety of cancer types (209, 212). Besides anti-neoplastic properties, MTX exerts potent anti-inflammatory actions (213). Several studies reported efficacy of low-dose MTX in the treatment of rheumatoid arthritis (RA) (214, 215). The compound has become a mainstay in therapy of RA and other autoimmune diseases, although its immunosuppressive mechanisms of action have not yet been fully clarified (213). Observational data revealed that low-dose MTX therapy associated with a lower risk for CVD and cardiovascular mortality (216, 217). The Cardiovascular Inflammation Reduction Trial (CIRT) included 4,786 patients with CAD and additional metabolic risk factors (type 2 diabetes or metabolic syndrome) randomly assigned to receive low-dose MTX or placebo (128). After a median follow-up of 2.3 years, MTX neither reduced pro-inflammatory biomarkers [IL-1β, IL-6, and C-reactive protein (CRP)] nor CVE. MTX was associated with modest elevations in liver enzymes, reductions in leukocyte counts and hematocrit levels, and an increased incidence of non-basal-cell skin cancers. Although the study had some methodical limitations (e.g., patients were not screened for an increased inflammatory risk), the data overall discouraged further investigations on MTX therapy for CVD prevention. In a recent murine study, nanoparticle-formulated MTX conferred substantial atheroprotection through modulating lipoprotein transcellular transport, reducing expression of pro-inflammatory cytokines and attenuating monocyte maturation and recruitment (129).

Although several immunotherapeutic approaches have yielded promising results in preclinical CVD models, only few of these strategies have proven beneficial in clinical studies (8, 218). Canakinumab (14), a monoclonal antibody targeting IL-1β, and colchicine (219, 220), an ancient drug traditionally used for gout therapy which exerts anti-inflammatory effects (among other potential mechanisms) through inhibition of the NOD-, LRR-, and pyrin domain-containing protein 3 (NLRP3) inflammasome in leukocytes (221), were demonstrated to reduce cardiovascular events in large-scale clinical trials. Although not targeting inflammatory pathways, monoclonal antibodies [evolocumab (13) and alirocumab (12)] or a siRNA-based compound [inclisiran (222, 223)] targeting PCSK9 to lower LDL cholesterol levels have proven clinically beneficial.

Considering that a pro-inflammatory immune response is critically involved in early elimination of mutated cells (68), immunotherapies exerting systemic anti-inflammatory effects may mitigate anti-tumor immunity and thus increase the risk of cancer development. In the following section we discuss recent evidence on the impact of above-mentioned immunotherapeutic strategies for CVD on cancer incidence (an overview is given in Table 2).

**Table 2:** Effects of anti-atherosclerotic immunotherapies (with clinically proven efficacy) on cancer.

**Type of immunotherapy**	**Target**	**Compounds**	**Effect on cancer in clinical trials**	**Effect on cancer in animal studies**	**Potential mechanisms**
Monoclonal antibodies	IL-1β	Canakinumab	↓ (224)	↓ (225)*	- Reduction of tumor-promoting chronic inflammation
	PCSK9	Alirocumab	–	↓ (226)	- Increased MHC I expression on tumor cell surface and enhanced tumor infiltration by cytotoxic T cells
		Evolocumab			
	CD3	Teplizumab	–	↓ (227)	- Induction of leukemic cell apoptosis
		Foralumab			
Natural anti-tubulin agent	NLRP3 inflammasome**	Colchicine	↓ (228)	↓ (229–232)	- Direct antiproliferative effects on tumor cells and induction of apoptosis - Enhancement of CD4^+^ and CD8^+^ T-cell-mediated anti-tumor immunity

↑ = pro-carcinogenic effect. ↓ = anti-carcinogenic effect. * In this study IL-1β knockout mice were shown to be protected from tumor development, but antibody-mediated IL-1β depletion was not investigated. **besides other anti-inflammatory mechanisms.

*IL-1β, interleukin 1 beta; NLRP3, NOD-, LRR-, and pyrin domain-containing protein 3; PCSK9, proprotein convertase subtilisin/kexin 9*.

### Canakinumab

Although canakinumab reduced CVE in patients with CAD, several limitations, such as a significant impairment of host defense and high costs, prevented its clinical approval for secondary prevention of CVD (14). Recent evidence suggests that IL-1β has a dual role in cancer development: On the one hand, it initiated pro-inflammatory anti-tumor immune responses by activating tumor antigen-specific T_H_1 and T_H_17 cells and facilitated tumor regression (233). On the other hand, IL-1β can promote carcinogenesis by inducing chronic inflammation, endothelial cell activation, angiogenesis, or development of immunosuppressive cells, such as tumor-associated macrophages and myeloid-derived suppressor cells (233). In contrast to anti-tumor immunity, which is critically involved in cancer elimination, chronic inflammation may drive tumorigenesis through several mechanisms including inhibition of antineoplastic immune responses, modulation of the tumor microenvironment to become more tumor-permissive, and direct tumor-promoting actions on epithelial and cancer cells (234). In line with this, IL-1β-deficient mice transplanted with melanoma cells were protected from development of local tumor and metastases (225) and canakinumab significantly reduced incidence of fatal cancer in the CANTOS trial (14). An exploratory analysis further revealed that canakinumab treatment especially reduced the incidence of lung cancer and lung cancer mortality (224). These findings motivated the initiation of three randomized phase III trials, CANOPY-A (NCT03447769), CANOPY-1 (NCT03631199), and CANOPY-2 (NCT03626545) (235), and a phase II trial, CANOPY-N (NCT03968419) (236) to investigate the potential of canakinumab in the treatment of non-small cell lung cancer (NSCLC). Although most trials are still ongoing, the CANOPY-2 study, which evaluated canakinumab in a second- or third-line treatment setting (that is in patients with locally advanced or metastatic NSCLC and tumor progression after or during platinum-based chemotherapy and PD-(L)1 inhibitor therapy) failed to meet the primary endpoint of overall survival (237).

### Colchicine

Colchicine is derived from the autumn crocus, which has been used for gout therapy since ancient times (238). Until today, colchicine represents a first-line drug for gout treatment (239). More recently, the compound has emerged as a promising candidate for secondary prevention of CVD. In two large-scale RCTs, the COLCOT (219) and LoDoCo 2 (220) trials, collectively enrolling more than 10,000 patients with recent myocardial infarction or chronic coronary syndrome, low-dose colchicine therapy significantly reduced the incidence of CVE. In contrast to canakinumab, colchicine did not increase the risk of fatal infections, although in COLCOT non-fatal pneumonia occurred more often in colchicine-treated patients. Nevertheless, colchicine therapy did not significantly reduce cardiovascular mortality in these trials and was associated with an almost significant increase in non-cardiovascular mortality in LoDoCo2 (220). A smaller RCT, enrolling 795 patients, reported a significantly higher rate of all-cause mortality (mainly due to non-cardiovascular deaths) in patients with acute coronary syndrome when colchicine was added to standard therapy (240). Recent meta-analyses confirmed that low-dose colchicine therapy in patients with CAD was associated with a significantly reduced risk of CVE (myocardial infarction, stroke, and the need for coronary revascularization) and a non-significant reduction of cardiovascular deaths, which was counterbalanced by a non-significant increase in non-cardiovascular deaths (241, 242). Considering the robust reduction of CVE observed in these studies as well as the low price and wide availability of the compound, low-dose colchicine might become an option for secondary prevention in high-risk CVD patients, but the increased non-cardiovascular death rates need further investigation.

Despite the increase in non-cardiovascular mortality, colchicine was not associated with higher rates of cancer in any of the above-mentioned studies (219, 220, 240). Preclinical evidence showed that colchicine may reduce tumor growth of several cancer types, such as prostate (229), hypopharyngeal (230) and liver cancer (231) as well as tumor implantation of pressure-activated colon carcinoma cells (232). The incidence of all-cause cancers was significantly reduced in 13,679 male gout patients (at least temporarily) treated with colchicine compared with 10,371 control gout patients (228). Besides reducing tumor cell proliferation and inducing apoptosis (243), colchicine enhances CD4^+^ and CD8^+^ T-cell-mediated anti-tumor immunity by promoting dendritic cell maturation and antigen presentation (244). Colchicine toxicity at high doses prevents its application as anti-tumor drug (243). Whether low-dose colchicine might confer clinically relevant anti-cancer effects has yet to be determined.

### PCSK9 Inhibitors

PCSK9, a protein which is primarily expressed in the liver, counteracts clearance of LDL cholesterol by inducing degradation of internalized LDL receptor in hepatocytes leading to increased plasma LDL cholesterol levels (245). Inhibition of PCSK9 has emerged as a highly effective second-line cholesterol lowering strategy, which has received class I recommendations by current guidelines (246). Two monoclonal antibodies targeting PCSK9, evolocumab (13) and alirocumab (12), and a siRNA-based compound, inclisiran (222, 223), are approved for clinical use in selected high-risk patients.

Cholesterol is a driving force in atherogenesis, yet its role in cancer is less clear: Both positive and negative correlations as well as absence of any associations between cholesterol levels and cancer development have been reported by clinical studies (247). Likewise, several meta-analyses concluded that statin therapy reduces cancer incidence or improves prognosis (248–250), whereas others found no such correlations (251, 252). PCSK9 inhibitors have not been reported to affect cancer incidence in clinical trials (12, 13). However, in a recent study PCSK9 inhibition could potentiate anti-tumor immune responses and thus substantially reduced tumor growth in murine cancer models (226), which was independent of cholesterol-lowering. PCSK9 induced lysosomal degradation of MHC-I and disrupted its recycling to the cell surface. PCSK9 inhibition, either through genetic deletion or administration of monoclonal antibodies, increased MHC-I expression on the tumor cell surface and thus enhanced tumor infiltration by cytotoxic T cells. Besides limiting tumor growth when administered alone, anti-PCSK9 antibodies significantly enhanced anti-tumor efficacy of ICI therapy (anti-PD-1) (226). Another recent study demonstrated that a nanoliposomal anti-PCSK9 vaccine limited tumor progression and improved survival in a murine model of colon carcinoma (253). Concluding, these preclinical data warrant further exploration of PCSK9 inhibitors as cancer therapeutics in clinical trials.

#### Anti-CD3 Antibody Therapy

Anti-CD3 monoclonal antibodies bind to the CD3-TCR complex on the surface of T cells and thus induce disappearance by shedding or internalization of the receptor complex (254). This process, which is termed antigenic modulation, renders T cells temporarily blind to their cognate antigen and leads to anergy or apoptosis of activated T cells (254). Anti-CD3 antibody therapy induced long-lasting T_reg_-mediated immune tolerance through increased TGF-β production by apoptotic T cells and phagocytes involved in clearance of apoptotic T cells (255, 256). Clinical application of the murine muromonab-CD3 is limited by high immunogenicity and resulting side effects (e.g., nausea, fever, headaches) (254). Humanized anti-CD3 antibodies (e.g., teplizumab, and otelixizumab) and a fully human anti-CD3 antibody (foralumab) have been developed, that were well-tolerated in initial clinical studies (254). Early clinical evidence suggests reasonable efficacy of such novel anti-CD3 antibodies in the treatment or prevention of autoimmune diseases, such as multiple sclerosis (257), type 1 diabetes (258–261), and inflammatory bowel disease (262). In several preclinical studies, intravenous or oral administration of anti-CD3 antibodies conferred substantial atheroprotection by enhancing TGF-β production and thereby inducing anti-atherogenic T_regs_ (263–265). Muromonab-CD3 was reported to significantly decrease CD3^+^ tumor cells in a patient with refractory T cell acute lymphoblastic leukemia (266), but was ineffective in enhancing immune activation in patients with solid tumors when administered in combination with high- or low-dose IL-2 (267, 268). However, a recent preclinical study demonstrated high anti-tumor efficacy of teplizumab and foralumab in murine models of T cell acute lymphoblastic leukemia (227). To date, the potential of humanized or fully human anti-CD3 antibodies in the treatment of CVD or cancer has not yet been investigated in clinical trials. Considering the promising data from animal studies these compounds merit further investigation into their clinical application.

## Adoptive T Cell Transfer in Cancer and Atherosclerosis

Chimeric antigen receptor (CAR) T cell therapy represents an innovative cancer treatment strategy, in which circulating T cells are isolated and genetically modified *in vitro* to express a synthetic tumor-antigen-specific receptor (269), which are subsequently expanded and infused back into the patient to attack tumor cells (269). In 2017, CAR T cells directed against CD19 (tisagenlecleucel and axicabtagene ciloleucel) showed substantial anti-tumor activity in patients with refractory large B cell lymphoma and follicular lymphoma (270, 271), which led to their FDA approval. Subsequently, anti-CD19 CAR T cells brexucabtagene autoleucel and lisocabtagene maraleucel were approved for treatment of mantle cell lymphoma and diffuse large B cell lymphoma, respectively (272, 273). Idecabtagene vicleucel targets B cell maturation antigen (BCMA) and is the first clinically approved CAR T cell therapy for multiple myeloma treatment (274). A major drawback of CAR T cell therapy is its association with severe and potentially fatal side effects (275). Cytokine-release syndrome (CRS), that can potentially develop into fulminant haemophagocytic lymphohistiocytosis (HLH), and CAR-T-cell-related encephalopathy syndrome (CRES), are the two most common adverse reactions (275). A recent study reported that CVE, such as new onset of heart failure or arrhythmias, occurred in 12% of 137 patients who received CAR T cell therapy (all events were associated with CRS) (276). Whether CAR T cells affect atherogenesis and increase the cardiovascular risk in the long-term, is unknown.

Adoptive transfer of autologous *ex vivo* expanded polyclonal T_regs_ has emerged as a promising strategy to treat autoimmune diseases and is currently investigated in clinical trials (277). First evidence suggested safety and efficacy of this approach for the treatment of type 1 diabetes (278, 279), prevention of graft-vs.-host-disease (280, 281), or transplant rejection (282). Therapeutic potency may be enhanced by utilization of antigen-specific rather than polyclonal T_regs_. Administration of ovalbumin-specific T_regs_, which respond to a major component of chicken egg white, was demonstrated to be safe and effective in patients with refractory Crohn's disease (283). Adoptive transfer of both polyclonal (284) and antigen-specific T_regs_ (responding to heat-shock protein 60) (285) conferred atheroprotection in *Apoe*^−/−^ mice fed with a WD for 6–8 weeks. Yet, administration of ApoB^+^ T_regs_ to WD-fed *Apoe*^−/−^ mice failed to limit plaque progression during a more extended period of observation (12 weeks) (43). In this study, more than half of all transferred cells lost expression of T_reg_ markers and converted into conventional T cells, which likely accounted for treatment failure. Clarification of the mechanisms underlying such phenotypic conversion and development of strategies to ensure T_reg_ stability are essential for clinical translation of this approach.

## Vaccination Strategies in the Treatment of Cancer and Atherosclerosis

Vaccination strategies aiming to induce pro-inflammatory immune responses against tumor-specific antigens or immune tolerance to plaque-associated autoantigens hold great promise for the treatment of cancer and atherosclerosis, respectively (4, 6). Through eliciting antigen-specific immunity, such approaches are highly effective without impairing the host defense against infectious agents and cancer cells or enhancing atherogenesis.

A series of animal studies have indicated the great potential of immunization against ApoB-related antigens for the treatment of atherosclerosis (6). The underlying idea originates from the observation that administration of oxLDL to hypercholesterolemic rabbits reduced atherosclerotic lesions (286). Subsequent studies identified an expansion of ApoB^+^ T_regs_ (42, 287, 288) and a humoral immune response against LDL (289) to account for the observed atheroprotection. Despite these promising preclinical data, several unknowns, such as optimal epitopes, adjuvants, administration route and vaccination scheme, stability of the atheroprotective immune response, and criteria for patient selection, have hitherto hindered translation of anti-atherosclerotic vaccination strategies into clinical practice (6). Recently, utilization of nanoparticle-formulated, nucleoside-modified messenger RNA (mRNA) without addition of adjuvants was demonstrated to enable sustained immune tolerance to MS-related autoantigens through induction of functional T_regs_ in mice (290). Considering that mRNA-based vaccines are already in clinical use, this approach has high translational potential for development of a vaccine against atherosclerosis.

Therapeutic cancer vaccines aim to induce a sustained effector immune-response against tumor-specific antigens (291). Initial approaches used self-antigens, which exist in non-malignant tissues, but are abnormally expressed or overexpressed by tumor cells, or applied non-self-antigens of viral origin (4, 291). Technological advances, such as next-generation sequencing, enabled identification of tumor-specific epitopes resulting from mutations (4, 291). These so-called neoepitopes or neoantigens are then evaluated for their capacity to bind human MHC-I or -II molecules. Candidates with high binding capacity can finally be utilized for development of personalized cancer vaccines or *in vitro* expansion of tumor-antigen specific CD4^+^ and CD8^+^ T cells, which are then transferred into the patient (4, 291). Vaccination with neoepitopes predicted to bind MHC-II or -I was shown to elicit strong CD4^+^ and/or CD8^+^ responses and thereby facilitate tumor rejection in animal models (292). In 2015, Carreno et al. firstly demonstrated the capability of a neoantigen-based vaccine strategy using dendritic cells as vaccine platform to induce a tumor-specific CD8^+^ T cell response in three patients with advanced melanoma (293). Subsequently peptide- and mRNA-based neoantigen vaccines were shown to induce strong CD4^+^ and CD8^+^ T cell responses alongside with significant tumor regression in melanoma patients (90, 91). Despite the promising results of these early clinical trials, several open questions on how to improve efficacy and feasibility of neoantigen-based tumor vaccines remain, that include identification of optimal antigens, delivery platforms, adjuvants, and routes of administration (4). Several clinical trials investigating the efficacy of neoantigen-based vaccine approaches in different cancer types are underway and will likely help to find answers to these questions (4, 291).

## Conclusion

Adaptive immunity is critically involved in the pathogenesis of atherosclerotic cardiovascular diseases and cancer, which represent the two most common causes of death worldwide. During the past few decades, novel treatment strategies enhancing anti-tumor immune responses have already found their way into clinical practice, whereas successful translation of strategies targeting atherogenesis-related immune responses into the clinic has not yet been accomplished. Given that some immunotherapies (e.g., CD20-, EGFR-, IL-1β- or PCSK9-targeting antibodies) were shown to protect from both cancer and atherosclerosis, inflammatory processes and immunity underlying carcinogenesis and atherogenesis may be closely interconnected. Further characterization of tumor-promoting and proatherogenic immune responses may help to identify novel pharmacological targets that allow simultaneous treatment of both disease entities. Further characterization of tumor-promoting and proatherogenic immune responses may help to identify novel pharmacological targets that allow simultaneous treatment of both disease entities. In that regard, multimodal sequencing approaches, such as Cellular Indexing of Transcriptomes and Epitopes by Sequencing (CITE-Seq), which allow combined measurement of gene and surface-protein expression on a single-cell level, will be valuable tools. Active immunization represents a novel, promising approach for the treatment of cancer and atherosclerosis. Preclinical studies have underscored the great anti-atherosclerotic potential of vaccination against plaque-related autoantigens. Further research is required to optimize this promising approach. Major objectives in this context include identification of optimal vaccine delivery platforms, adjuvants and administration routes. Furthermore, development of clinically feasible approaches to identify eligible patients, to determine expression of target antigens within an individual patient, and to monitor treatment responses will be crucial for broad implementation of this approach into clinical practice. If these obstacles can be overcome, active immunization may prospectively take cancer and atherosclerosis therapy to the next level.

## Author Contributions

FN wrote the manuscript. FP prepared the figure. FN and HW conceptualized the work. HW and FH supervised the work and provided funding. All authors substantially contributed to data research, critically discussed the content, reviewed the manuscript before submission, and have read and agreed to the published version of the manuscript.

## Funding

This research was funded by the Deutsche Forschungsgemeinschaft [SFB TRR259 (397484323) and CCRC GRK2407 (360043781) to HW and HO 5279/2-1 to FH], the Neven-DuMont foundation (to HW), and the Koeln Fortune Program (363/2020 to FN).

## Conflict of Interest

The authors declare that the research was conducted in the absence of any commercial or financial relationships that could be construed as a potential conflict of interest.

## Publisher's Note

All claims expressed in this article are solely those of the authors and do not necessarily represent those of their affiliated organizations, or those of the publisher, the editors and the reviewers. Any product that may be evaluated in this article, or claim that may be made by its manufacturer, is not guaranteed or endorsed by the publisher.

## References

[1] Roth GA Abate D Abate KH Abay SM Abbafati C Abbasi N. Global, regional, and national age-sex-specific mortality for 282 causes of death in 195 countries and territories, 1980–2017: a systematic analysis for the Global Burden of Disease Study 2017. Lancet. (2018) 392:1736–88. 10.1016/S0140-6736(18)32203-7 30496103 PMC6227606

[2] Balkwill F Mantovani A. Inflammation and cancer: back to Virchow? Lancet. (2001) 357:539–45. 10.1016/S0140-6736(00)04046-0 11229684

[3] Libby P. Inflammation in atherosclerosis. Arterioscler Thromb Vasc Biol. (2012) 32:2045–51. 10.1161/ATVBAHA.108.179705 22895665 PMC3422754

[4] Blass E Ott PA. Advances in the development of personalized neoantigen-based therapeutic cancer vaccines. Nat Rev Clin Oncol. (2021) 18:215–29. 10.1038/s41571-020-00460-2 33473220 PMC7816749

[5] Ait-Oufella H Lavillegrand J-R Tedgui A. Regulatory T cell-enhancing therapies to treat atherosclerosis. Cells. (2021) 10:723. 10.3390/cells10040723 33805071 PMC8064079

[6] Nettersheim FS De Vore L Winkels H. Vaccination in atherosclerosis. Cells. (2020) 9:2560. 10.3390/cells9122560 33266027 PMC7760548

[7] Waldman AD Fritz JM Lenardo MJ. A guide to cancer immunotherapy: from T cell basic science to clinical practice. Nat Rev Immunol. (2020) 20:651–668. 10.1038/s41577-020-0306-5 32433532 PMC7238960

[8] Zhao TX Mallat Z. Targeting the immune system in atherosclerosis: JACC state-of-the-art review. J Am Coll Cardiol. (2019) 73:1691–706. 10.1016/j.jacc.2018.12.083 30947923

[9] Dobosz P Dzieciatkowski T. The intriguing history of cancer immunotherapy. Front Immunol. (2019) 10:2965. 10.3389/fimmu.2019.02965 31921205 PMC6928196

[10] Libby P. The changing landscape of atherosclerosis. Nature. (2021) 592:524–33. 10.1038/s41586-021-03392-8 33883728

[11] Dall'Olio FG Marabelle A Caramella C Garcia C Aldea M Chaput N. Tumour burden and efficacy of immune-checkpoint inhibitors. Nat Rev Clin Oncol. (2021) 2021:1–16. 10.1038/s41571-021-00564-3 34642484

[12] Schwartz GG Steg PG Szarek M Bhatt DL Bittner VA Diaz R. Alirocumab and cardiovascular outcomes after acute coronary syndrome. N Engl J Med. (2018) 379:2097–107. 10.1056/NEJMoa1801174 30403574

[13] Sabatine MS Giugliano RP Keech AC Honarpour N Wiviott SD Murphy SA. Evolocumab and clinical outcomes in patients with cardiovascular disease. N Engl J Med. (2017) 376:1713–22. 10.1056/NEJMoa1615664 28304224

[14] Ridker PM Everett BM Thuren T MacFadyen JG Chang WH Ballantyne C. Antiinflammatory therapy with canakinumab for atherosclerotic disease. N Engl J Med. (2017) 377:1119–31. 10.1056/NEJMoa1707914 28845751

[15] Libby P. Targeting inflammatory pathways in cardiovascular disease: the inflammasome, interleukin-1, interleukin-6 and beyond. Cells. (2021) 10:951. 10.3390/cells10040951 33924019 PMC8073599

[16] Lobenwein D Kocher F Dobner S Gollmann-Tepeköylü C Holfeld J. Cardiotoxic mechanisms of cancer immunotherapy – a systematic review. Int J Cardiol. (2021) 323:179–87. 10.1016/j.ijcard.2020.08.033 32800915

[17] Drobni ZD Alvi RM Taron J Zafar A Murphy SP Rambarat PK. Association between immune checkpoint inhibitors with cardiovascular events and atherosclerotic plaque. Circulation. (2020) 142:2299–311. 10.1161/CIRCULATIONAHA.120.049981 33003973 PMC7736526

[18] Arnold M Rutherford MJ Bardot A Ferlay J Andersson TML Myklebust TÅ. Progress in cancer survival, mortality, and incidence in seven high-income countries 1995–2014 (ICBP SURVMARK-2): a population-based study. Lancet Oncol. (2019) 20:1493–505. 10.1016/S1470-2045(19)30456-5 31521509 PMC6838671

[19] Stoltzfus KC Zhang Y Sturgeon K Sinoway LI Trifiletti DM Chinchilli VM. Fatal heart disease among cancer patients. Nat Commun. (2020) 11:1–8. 10.1038/s41467-020-15639-5 32332714 PMC7181822

[20] Sturgeon KM Deng L Bluethmann SM Zhou S Trifiletti DM Jiang C. A population-based study of cardiovascular disease mortality risk in US cancer patients. Eur Heart J. (2019) 40:3889–97. 10.1093/eurheartj/ehz766 31761945 PMC6925383

[21] Wang L Wang F Chen L Geng Y Yu S Chen Z. Long-term cardiovascular disease mortality among 160 834 5-year survivors of adolescent and young adult cancer: an American population-based cohort study. Eur Heart J. (2020) 42:101–9. 10.1093/eurheartj/ehaa779 33156911

[22] Strongman H Gadd S Matthews A Mansfield KE Stanway S Lyon AR. Medium and long-term risks of specific cardiovascular diseases in survivors of 20 adult cancers: a population-based cohort study using multiple linked UK electronic health records databases. Lancet. (2019) 394:1041–54. 10.1016/S0140-6736(19)31674-5 31443926 PMC6857444

[23] Sung H Ferlay J Siegel RL Laversanne M Soerjomataram I Jemal A. Global cancer statistics 2020: GLOBOCAN estimates of incidence and mortality worldwide for 36 cancers in 185 countries. CA Cancer J Clin. (2021) 71:209–49. 10.3322/caac.21660 33538338

[24] Wolf D Ley K. Immunity and inflammation in atherosclerosis. Circ Res. (2019) 124:315–27. 10.1161/CIRCRESAHA.118.313591 30653442 PMC6342482

[25] Bentzon JF Otsuka F Virmani R Falk E. Mechanisms of plaque formation and rupture. Circ Res. (2014) 114:1852–66. 10.1161/CIRCRESAHA.114.302721 24902970

[26] Wentzel JJ Chatzizisis YS Gijsen FJH Giannoglou GD Feldman CL Stone PH. Endothelial shear stress in the evolution of coronary atherosclerotic plaque and vascular remodelling: current understanding and remaining questions. Cardiovasc Res. (2012) 96:234–43. 10.1093/cvr/cvs217 22752349

[27] Roy P Orecchioni M Ley K. How the immune system shapes atherosclerosis: roles of innate and adaptive immunity. Nat Rev Immunol. (2021). 10.1038/s41577-021-00584-1. [Epub ahead of print]. PMC10111155 34389841

[28] Shah DK Zúñiga-Pflücker JC. An overview of the intrathymic intricacies of T cell development. J Immunol. (2014) 192:4017–23. 10.4049/jimmunol.1302259 24748636

[29] Klein L Kyewski B Allen PM Hogquist KA. Positive and negative selection of the T cell repertoire: what thymocytes see (and don't see). Nat Rev Immunol. (2014) 14:377–91. 10.1038/nri3667 24830344 PMC4757912

[30] Van Den Broek T Borghans JAM Van Wijk F. The full spectrum of human naive T cells. Nat Rev Immunol. (2018) 18:363–73. 10.1038/s41577-018-0001-y 29520044

[31] Neefjes J Jongsma MLM Paul P Bakke O. Towards a systems understanding of MHC class i and MHC class II antigen presentation. Nat Rev Immunol. (2011) 11:823–36. 10.1038/nri3084 22076556

[32] Smith-Garvin JE Koretzky GA Jordan MS. T cell activation. Annu Rev Immunol. (2009) 27:591–619. 10.1146/annurev.immunol.021908.132706 19132916 PMC2740335

[33] Zhou L Chong MMW Littman DR. Plasticity of CD4+ T cell lineage differentiation. Immunity. (2009) 30:646–55. 10.1016/j.immuni.2009.05.001 19464987

[34] Saigusa R Winkels H Ley K. T cell subsets and functions in atherosclerosis. Nat Rev Cardiol. (2020) 17:387–401. 10.1038/s41569-020-0352-5 32203286 PMC7872210

[35] Jonasson L Holm J Skalli O Bondjers G Hansson GK. Regional accumulations of T cells, macrophages, and smooth muscle cells in the human atherosclerotic plaque. Arteriosclerosis. (1986) 6:131–8. 10.1161/01.ATV.6.2.131 2937395

[36] Grivel JC Ivanova O Pinegina N Blank PS Shpektor A Margolis LB. Activation of T lymphocytes in atherosclerotic plaques. Arterioscler Thromb Vasc Biol. (2011) 31:2929–37. 10.1161/ATVBAHA.111.237081 21960562 PMC3401061

[37] Stemme S Faber B Holm J Wiklund O Witztum JL Hansson GK. T lymphocytes from human atherosclerotic plaques recognize oxidized low density lipoprotein. Proc Natl Acad Sci USA. (1995) 92:3893–7. 10.1073/pnas.92.9.3893 7732003 PMC42068

[38] Ma SD Mussbacher M Galkina E V. Functional role of B cells in atherosclerosis. Cells. (2021) 10:270. 10.3390/cells10020270 33572939 PMC7911276

[39] Schäfer S Zernecke A. CD8+ T cells in atherosclerosis. Cells. (2020) 10:37. 10.3390/cells10010037 33383733 PMC7823404

[40] Tse K Gonen A Sidney J Ouyang H Witztum JL Sette A. Atheroprotective vaccination with MHC-II restricted peptides from ApoB-100. Front Immunol. (2013) 4:493. 10.3389/fimmu.2013.00493 24416033 PMC3873602

[41] Kimura T Tse K McArdle S Gerhardt T Miller J Mikulski Z. Atheroprotective vaccination with MHC-II-restricted ApoB peptides induces peritoneal IL-10-producing CD4 T cells. Am J Physiol Hear Circ Physiol. (2017) 312:H781–90. 10.1152/ajpheart.00798.2016 28087520 PMC5407161

[42] Kimura T Kobiyama K Winkels H Tse K Miller J Vassallo M. Regulatory CD4+ T cells recognize major histocompatibility complex class II molecule-restricted peptide epitopes of apolipoprotein B. Circulation. (2018) 138:1130–43. 10.1161/CIRCULATIONAHA.117.031420 29588316 PMC6160361

[43] Wolf D Gerhardt T Winkels H Anto Michel N Pramod AB Ghosheh Y. Pathogenic autoimmunity in atherosclerosis evolves from initially protective ApoB-reactive CD4 ^+^ T-regulatory cells. Circulation. (2020) 142:1279–93. 10.1161/CIRCULATIONAHA.119.042863 32703007 PMC7515473

[44] Gaddis DE Padgett LE Wu R McSkimming C Romines V Taylor AM. Apolipoprotein AI prevents regulatory to follicular helper T cell switching during atherosclerosis. Nat Commun. (2018) 9:1095. 10.1038/s41467-018-03493-5 29545616 PMC5854619

[45] Butcher MJ Filipowicz AR Waseem TC McGary CM Crow KJ Magilnick N. Atherosclerosis-driven treg plasticity results in formation of a dysfunctional subset of plastic IFNγ+ Th1/tregs. Circ Res. (2016) 119:1190–203. 10.1161/CIRCRESAHA.116.309764 27635087 PMC5242312

[46] Klingenberg R Gerdes N Badeau RM Gisterå A Strodthoff D Ketelhuth DFJ. Depletion of FOXP3+ regulatory T cells promotes hypercholesterolemia and atherosclerosis. J Clin Invest. (2013) 123:1323–34. 10.1172/JCI63891 23426179 PMC3582120

[47] Sage AP Tsiantoulas D Binder CJ Mallat Z. The role of B cells in atherosclerosis. Nat Rev Cardiol. (2019) 16:180–96. 10.1038/s41569-018-0106-9 30410107

[48] Yla-Herttuala S Palinski W Butler SW Picard S Steinberg D Witztum JL. Rabbit and human atherosclerotic lesions contain IgG that recognizes epitopes of oxidized LDL. Arterioscler Thromb. (1994) 14:32–40. 10.1161/01.ATV.14.1.32 7506053

[49] Orekhov AN Tertov V V., Kabakov AE, Adamova IY, Pokrovsky SN, Smirnov VN. Autoantibodies against modified low density lipoprotein. Nonlipid factor of blood plasma that stimulates foam cell formation. Arterioscler Thromb. (1991) 11:316–26. 10.1161/01.ATV.11.2.316 1998649

[50] Parums D V., Brown DL, Mitchinson MJ. Serum antibodies to oxidized low-density lipoprotein and ceroid in chronic periaortitis. Arch Pathol Lab Med. (1990) 114:383–387. 2322097

[51] Zernecke A Winkels H Cochain C Williams JW Wolf D Soehnlein O. Meta-analysis of leukocyte diversity in atherosclerotic mouse aortas. Circ Res. (2020) 127:402–26. 10.1161/CIRCRESAHA.120.316903 32673538 PMC7371244

[52] Shaw PX Hörkkö S Tsimikas S Chang MK Palinski W Silverman GJ. Human-derived anti-oxidized LDL autoantibody blocks uptake of oxidized LDL by macrophages and localizes to atherosclerotic lesions *in vivo*. Arterioscler Thromb Vasc Biol. (2001) 21:1333–9. 10.1161/hq0801.093587 11498462

[53] Hörkkö S Bird DA Miller E Itabe H Leitinger N Subbanagounder G. Monoclonal autoantibodies specific for oxidized phospholipids or oxidized phospholipid–protein adducts inhibit macrophage uptake of oxidized low-density lipoproteins. J Clin Invest. (1999) 103:117–28. 10.1172/JCI4533 9884341 PMC407862

[54] Que X Hung M-Y Yeang C Gonen A Prohaska TA Sun X. Oxidized phospholipids are proinflammatory and proatherogenic in hypercholesterolaemic mice. Nature. (2018) 558:301–6. 10.1038/s41586-018-0198-8 29875409 PMC6033669

[55] Major AS Fazio S Linton MF. B-lymphocyte deficiency increases atherosclerosis in LDL receptor-null mice. Arterioscler Thromb Vasc Biol. (2002) 22:1892–8. 10.1161/01.ATV.0000039169.47943.EE 12426221

[56] Ait-Oufella H Herbin O Bouaziz JD Binder CJ Uyttenhove C Laurans L. B cell depletion reduces the development of atherosclerosis in mice. J Exp Med. (2010) 207:1579–87. 10.1084/jem.20100155 20603314 PMC2916123

[57] Kyaw T Tay C Khan A Dumouchel V Cao A To K. Conventional B2 B cell depletion ameliorates whereas its adoptive transfer aggravates atherosclerosis. J Immunol. (2010) 185:4410–19. 10.4049/jimmunol.1000033 20817865

[58] Cyster JG Allen CDC. B cell responses: cell interaction dynamics and decisions. Cell. (2019) 177:524–40. 10.1016/j.cell.2019.03.016 31002794 PMC6538279

[59] Baumgarth N. The double life of a B-1 cell: self-reactivity selects for protective effector functions. Nat Rev Immunol. (2011) 11:34–46. 10.1038/nri2901 21151033

[60] Oiseth SJ Aziz MS. Cancer immunotherapy: a brief review of the history, possibilities, and challenges ahead. J Cancer Metastasis Treat. (2017) 3:250–61. 10.20517/2394-4722.2017.41

[61] Busch W. Aus der Sitzung der medicinischen section vom 13 november 1867. Berlin Klin Wochenschr. (1868) 5:137.

[62] Fehleisen F. Ueber die Züchtung der Erysipelkokken auf künstlichem Nährboden und ihre Uebertragbarkeit auf den Menschen. Dtsch Med Wochenschr. (1882) 8:553–4. 10.1055/s-0029-119680626606158

[63] Coley WB. The treatment of inoperable sarcoma by bacterial toxins (the mixed toxins of the streptococcus erysipelas and the Bacillus prodigiosus). Proc R Soc Med. (1910) 3:1. 10.1177/003591571000301601 19974799 PMC1961042

[64] Ehrlich P. Ueber den jetzigen Stand der Karzinomforschung. Ned Tijdschr Geneeskd. (1909) 53:273–90.

[65] Gross L. Intradermal immunization of C3H mice against a sarcoma that originated in an animal of the same line. Cancer Res. (1943) 3:326–33.

[66] Foley EJ. Antigenic properties of methylcholanthrene-induced tumors in mice of the strain of origin. Cancer Res. (1953) 13:835–7. 13116120

[67] Ribatti D. The concept of immune surveillance against tumors: the first theories. Oncotarget. (2017) 8:7175. 10.18632/oncotarget.12739 27764780 PMC5351698

[68] Dunn GP Bruce AT Ikeda H Old LJ Schreiber RD. Cancer immunoediting: from immunosurveillance to tumor escape. Nat Immunol. (2002) 3:991–8. 10.1038/ni1102-991 12407406

[69] Stutman O. Tumor development after 3-methylcholanthrene in immunologically deficient athymic-nude mice. Science. (1974) 183:534–6. 10.1126/science.183.4124.534 4588620

[70] Shankaran V Ikeda H Bruce AT White JM Swanson PE Old LJ. IFNγ and lymphocytes prevent primary tumour development and shape tumour immunogenicity. Nature. (2001) 410:1107–11. 10.1038/35074122 11323675

[71] Street SEA Cretney E Smyth MJ. Perforin and interferon-γ activities independently control tumor initiation, growth, and metastasis. Blood. (2001) 97:192–7. 10.1182/blood.V97.1.192 11133760

[72] Street SEA Trapani JA MacGregor D Smyth MJ. Suppression of lymphoma and epithelial malignancies effected by interferon γ. J Exp Med. (2002) 196:129. 10.1084/jem.20020063 12093877 PMC2194011

[73] Kaplan DH Shankaran V Dighe AS Stockert E Aguet M Old LJ. Demonstration of an interferon γ-dependent tumor surveillance system in immunocompetent mice. Proc Natl Acad Sci. (1998) 95:7556–61. 10.1073/pnas.95.13.7556 9636188 PMC22681

[74] Pham SM Kormos RL Landreneau RJ Kawai A Gonzalez-Cancel I Hardesty RL. Solid tumors after heart transplantation: lethality of lung cancer. Ann Thorac Surg. (1995) 60:1623–6. 10.1016/0003-4975(95)00120-4 8787454

[75] Penn I. Malignant melanoma in organ allograft recipients. Transplantation. (1996) 61:274–8. 10.1097/00007890-199601270-00019 8600636

[76] Birkeland SA Storm HH Lamm LU Barlow L Blohmé I Forsberg B. Cancer risk after renal transplantation in the nordic countries, 1964–1986. Int J Cancer. (1995) 60:183–9. 10.1002/ijc.2910600209 7829213

[77] Clark WH Elder DE Guerry D Braitman LE Trock BJ Schultz D. Model predicting survival in stage I melanoma based on tumor progression. JNCI J Natl Cancer Inst. (1989) 81:1893–904. 10.1093/jnci/81.24.1893 2593166

[78] Clemente CG Mihm Jr. MC, Bufalino R, Zurrida S, Collini P, Cascinelli N. Prognostic value of tumor infiltrating lymphocytes in the vertical growth phase of primary cutaneous melanoma. Cancer. (1996) 77:1303–10. 8608507 10.1002/(SICI)1097-0142(19960401)77:7<1303::AID-CNCR12>3.0.CO;2-5

[79] Smyth MJ Thia KYT Street SEA Cretney E Trapani JA Taniguchi M. Differential tumor surveillance by Natural Killer (Nk) and Nkt cells. J Exp Med. (2000) 191:661–8. 10.1084/jem.191.4.661 10684858 PMC2195840

[80] Svane IM Engel A-M Nielsen M-B Ljunggren H-G Rygaard J Werdelin O. Chemically induced sarcomas from nude mice are more immunogenic than similar sarcomas from congenic normal mice. Eur J Immunol. (1996) 26:1844–50. 10.1002/eji.1830260827 8765030

[81] Fridman WH. From cancer immune surveillance to cancer immunoediting: birth of modern immuno-oncology. J Immunol. (2018) 201:825–6. 10.4049/jimmunol.1800827 30038034

[82] Schumacher TN Schreiber RD. Neoantigens in cancer immunotherapy. Science. (2015) 348:69–74. 10.1126/science.aaa4971 25838375

[83] O'Donnell JS Teng MWL Smyth MJ. Cancer immunoediting and resistance to T cell-based immunotherapy. Nat Rev Clin Oncol. (2018) 16:151–67. 10.1038/s41571-018-0142-8 30523282

[84] Castle JC Kreiter S Diekmann J Löwer M Roemer N van de Graaf J de. Exploiting the mutanome for tumor vaccination. Cancer Res. (2012) 72:1081–91. 10.1158/0008-5472.CAN-11-3722 22237626

[85] Matsushita H Vesely MD Koboldt DC Rickert CG Uppaluri R Magrini VJ. Cancer exome analysis reveals a T-cell-dependent mechanism of cancer immunoediting. Nature. (2012) 482:400–4. 10.1038/nature10755 22318521 PMC3874809

[86] Kreiter S Vormehr M van de Roemer N Diken M Löwer M Diekmann J. Mutant MHC class II epitopes drive therapeutic immune responses to cancer. Nature. (2015) 520:692–6. 10.1038/nature14426 25901682 PMC4838069

[87] Robbins PF Lu Y-C El-Gamil M Li YF Gross C Gartner J. Mining exomic sequencing data to identify mutated antigens recognized by adoptively transferred tumor-reactive T cells. Nat Med. (2013) 19:747–52. 10.1038/nm.3161 23644516 PMC3757932

[88] Balachandran VP Łuksza M Zhao JN Makarov V Moral JA Remark R Herbst B. Identification of unique neoantigen qualities in long-term survivors of pancreatic cancer. Nature. (2017) 551:512–6. 10.1038/nature24462 29132146 PMC6145146

[89] Linnemann C van Buuren MM Bies L Verdegaal EME Schotte R Calis JJA. High-throughput epitope discovery reveals frequent recognition of neo-antigens by CD4+ T cells in human melanoma. Nat Med. (2014) 21:81–5. 10.1038/nm.3773 25531942

[90] Sahin U Derhovanessian E Miller M Kloke B-P Simon P Löwer M. Personalized RNA mutanome vaccines mobilize poly-specific therapeutic immunity against cancer. Nature. (2017) 547:222–6. 10.1038/nature23003 28678784

[91] Ott PA Hu Z Keskin DB Shukla SA Sun J Bozym DJ. An immunogenic personal neoantigen vaccine for patients with melanoma. Nature. (2017) 547:217–21. 10.1038/nature22991 28678778 PMC5577644

[92] Alspach E Lussier DM Miceli AP Kizhvatov I DuPage M Luoma AM. MHC-II neoantigens shape tumour immunity and response to immunotherapy. Nature. (2019) 574:696–701. 10.1038/s41586-019-1671-8 31645760 PMC6858572

[93] Lennerz V Fatho M Gentilini C Frye RA Lifke A Ferel D. The response of autologous T cells to a human melanoma is dominated by mutated neoantigens. Proc Natl Acad Sci. (2005) 102:16013–18. 10.1073/pnas.0500090102 16247014 PMC1266037

[94] Brightman SE Naradikian MS Miller AM Schoenberger SP. Harnessing neoantigen specific CD4 T cells for cancer immunotherapy. J Leukoc Biol. (2020) 107:625–33. 10.1002/JLB.5RI0220-603RR 32170883 PMC7793607

[95] Zander R Schauder D Xin G Nguyen C Wu X Zajac A. CD4+ T cell help is required for the formation of a cytolytic CD8+ T cell subset that protects against chronic infection and cancer. Immunity. (2019) 51:1028–42.e4. 10.1016/j.immuni.2019.10.009 31810883 PMC6929322

[96] Schoenberger SP Toes REM van der Voort EIH Offringa R Melief CJM. T-cell help for cytotoxic T lymphocytes is mediated by CD40–CD40L interactions. Nature. (1998) 393:480–3. 10.1038/31002 9624005

[97] Tay RE Richardson EK Toh HC. Revisiting the role of CD4+ T cells in cancer immunotherapy—new insights into old paradigms. Cancer Gene Ther. (2020) 28:5–17. 10.1038/s41417-020-0183-x 32457487 PMC7886651

[98] Tran E Turcotte S Gros A Robbins PF Lu YC Dudley ME. Cancer immunotherapy based on mutation-specific CD4+ T cells in a patient with epithelial cancer. Science. (2014) 344:641–5. 10.1126/science.1251102 24812403 PMC6686185

[99] Laheurte C Dosset M Vernerey D Boullerot L Gaugler B Gravelin E. Distinct prognostic value of circulating anti-telomerase CD4+ Th1 immunity and exhausted PD-1+/TIM-3+ T cells in lung cancer. Br J Cancer. (2019) 121:405–16. 10.1038/s41416-019-0531-5 31358938 PMC6738094

[100] Sayour EJ McLendon P McLendon R De Leon G Reynolds R Kresak J. Increased proportion of FoxP3+ regulatory T cells in tumor infiltrating lymphocytes is associated with tumor recurrence and reduced survival in patients with glioblastoma. Cancer Immunol Immunother. (2015) 64:419–27. 10.1007/s00262-014-1651-7 25555571 PMC4774199

[101] Nishikawa H Sakaguchi S. Regulatory T cells in cancer immunotherapy. Curr Opin Immunol. (2014) 27:1–7. 10.1016/j.coi.2013.12.005 24413387

[102] Valzasina B Piconese S Guiducci C Colombo MP. Tumor-induced expansion of regulatory T cells by conversion of CD4+CD25– lymphocytes is thymus and proliferation independent. Cancer Res. (2006) 66:4488–95. 10.1158/0008-5472.CAN-05-4217 16618776

[103] Bonertz A Weitz J Pietsch D-HK Rahbari NN Schlude C Ge Y. Antigen-specific Tregs control T cell responses against a limited repertoire of tumor antigens in patients with colorectal carcinoma. J Clin Invest. (2009) 119:3311–21. 10.1172/JCI39608 19809157 PMC2769188

[104] Schreiber S Hammers CM Kaasch AJ Schraven B Dudeck A Kahlfuss S. Metabolic interdependency of Th2 cell-mediated type 2 immunity and the tumor microenvironment. Front Immunol. (2021) 12:632581. 10.3389/fimmu.2021.632581 34135885 PMC8201396

[105] Li T Wu B Yang T Zhang L Jin K. The outstanding antitumor capacity of CD4+ T helper lymphocytes. Biochim Biophys Acta Rev Cancer. (2020) 1874:188439. 10.1016/j.bbcan.2020.188439 32980465

[106] Calabretta R Hoeller C Pichler V Mitterhauser M Karanikas G Haug A. Immune checkpoint inhibitor therapy induces inflammatory activity in large arteries. Circulation. (2020) 142:2396–8. 10.1161/CIRCULATIONAHA.120.048708 32894978

[107] Bu D Tarrio M Maganto-Garcia E Stavrakis G Tajima G Lederer J. Impairment of the programmed cell death-1 pathway increases atherosclerotic lesion development and inflammation. Arterioscler Thromb Vasc Biol. (2011) 31:1100–7. 10.1161/ATVBAHA.111.224709 21393583 PMC3104026

[108] Gotsman I Grabie N Dacosta R Sukhova G Sharpe A Lichtman AH. Proatherogenic immune responses are regulated by the PD-1/PD-L pathway in mice. J Clin Invest. (2007) 117:2974–82. 10.1172/JCI31344 17853943 PMC1974866

[109] Poels K Leent MMT van Reiche ME Kusters PJH Huveneers S Winther MPJ de. Antibody-mediated inhibition of CTLA4 aggravates atherosclerotic plaque inflammation and progression in hyperlipidemic mice. Cells. (2020) 9:1987. 10.3390/cells9091987 32872393 PMC7565685

[110] Poels K van Leent MMT Boutros C Tissot H Roy S Meerwaldt AE. Immune checkpoint inhibitor therapy aggravates T cell–driven plaque inflammation in atherosclerosis. JACC CardioOncology. (2020) 2:599–610. 10.1016/j.jaccao.2020.08.007 34396271 PMC8352210

[111] Cochain C Chaudhari SM Koch M Wiendl H Eckstein H-H Zernecke A. Programmed cell death-1 deficiency exacerbates T cell activation and atherogenesis despite expansion of regulatory T cells in atherosclerosis-prone mice. PLoS ONE. (2014) 9:e93280. 10.1371/journal.pone.0093280 24691202 PMC3972211

[112] Kim DG Lee J Seo WJ Lee JG Kim BS Kim MS. Rituximab protects against development of atherosclerotic cardiovascular disease after kidney transplantation: a propensity-matched study. Sci Rep. (2019) 9:1–8. 10.1038/s41598-019-52942-8 31712593 PMC6848081

[113] Novikova DS Popkova T V., Lukina G V., Luchikhina EL, Karateev DE, Volkov A V., et al. The effects of rituximab on lipids, arterial stiffness and carotid intima-media thickness in rheumatoid arthritis. J Korean Med Sci. (2016) 31:202–7. 10.3346/jkms.2016.31.2.202 26839473 PMC4729499

[114] Hsue PY Scherzer R Grunfeld C Imboden J Wu Y Puerto G del. Depletion of B-cells with rituximab improves endothelial function and reduces inflammation among individuals with rheumatoid arthritis. J Am Heart Assoc. (2014) 3:e001278. 10.1161/JAHA.114.001267 25336464 PMC4323827

[115] Totzeck M Mincu RI Rassaf T. Cardiovascular adverse events in patients with cancer treated with bevacizumab: a meta-analysis of more than 20 000 patients. J Am Heart Assoc. (2017) 6:e006278. 10.1161/JAHA.117.006278 28862931 PMC5586462

[116] Schutz FAB Je Y Azzi GR Nguyen PL Choueiri TK. Bevacizumab increases the risk of arterial ischemia: a large study in cancer patients with a focus on different subgroup outcomes. Ann Oncol. (2011) 22:1404–12. 10.1093/annonc/mdq587 21115602

[117] Chen X-L Lei Y-H Liu C-F Yang Q-F Zuo P-Y Liu C-Y. Angiogenesis inhibitor bevacizumab increases the risk of ischemic heart disease associated with chemotherapy: a meta-analysis. PLoS ONE. (2013) 8:e66721. 10.1371/journal.pone.0066721 23818962 PMC3688569

[118] Winnik S Lohmann C Siciliani G Lukowicz T von Kuschnerus K Kraenkel N. Systemic VEGF inhibition accelerates experimental atherosclerosis and disrupts endothelial homeostasis – implications for cardiovascular safety. Int J Cardiol. (2013) 168:2453–61. 10.1016/j.ijcard.2013.03.010 23561917

[119] Zeboudj L Giraud A Guyonnet L Zhang Y Laurans L Esposito B. Selective EGFR (Epidermal Growth Factor Receptor) deletion in myeloid cells limits atherosclerosis—brief report. Arterioscler Thromb Vasc Biol. (2018) 38:114–19. 10.1161/ATVBAHA.117.309927 29191921

[120] Zeboudj L Maître M Guyonnet L Laurans L Joffre J Lemarie J. Selective EGF-receptor inhibition in CD4+ T cells induces anergy and limits atherosclerosis. J Am Coll Cardiol. (2018) 71:160–72. 10.1016/j.jacc.2017.10.084 29325640

[121] Wang L Huang Z Huang W Chen X Shan P Zhong P. Inhibition of epidermal growth factor receptor attenuates atherosclerosis via decreasing inflammation and oxidative stress. Sci Rep. (2017) 7:1–14. 10.1038/srep45917 28374780 PMC5379239

[122] Somers EC Zhao W Lewis EE Wang L Wing JJ Sundaram B. Type I interferons are associated with subclinical markers of cardiovascular disease in a cohort of systemic lupus erythematosus patients. PLoS ONE. (2012) 7:e37000. 10.1371/journal.pone.0037000 22606325 PMC3351452

[123] Santinelli L Girolamo G De Borrazzo C Vassalini P Pinacchio C Cavallari EN. Alteration of type I interferon response is associated with subclinical atherosclerosis in virologically suppressed HIV-1-infected male patients. J Med Virol. (2021) 93:4930–8. 10.1002/jmv.27028 33913525 PMC8360015

[124] Levy Z Rachmani R Trestman S Dvir A Shaish A Ravid M. Low-dose interferon-α accelerates atherosclerosis in an LDL receptor-deficient mouse model. Eur J Intern Med. (2003) 14:479–83. 10.1016/j.ejim.2003.08.010 14962699

[125] Lee RE Lotze MT Skibber JM Tucker E Bonow RO Ognibene FP. Cardiorespiratory effects of immunotherapy with interleukin-2. J Clin Oncol. (1989) 7:7–20. 10.1200/JCO.1989.7.1.7 2783338

[126] Upadhya S Mooteri S Peckham N Pai RG. Atherogenic Effect of interleukin-2 and antiatherogenic effect of interleukin-2 antibody in apo-E-deficient mice. Angiology. (2016) 55:289–94. 10.1177/000331970405500308 15156262

[127] Dinh TN Kyaw TS Kanellakis P To K Tipping P Toh B-H. Cytokine therapy with interleukin-2/anti–interleukin-2 monoclonal antibody complexes expands CD4+CD25+Foxp3+ regulatory T cells and attenuates development and progression of atherosclerosis. Circulation. (2012) 126:1256–66. 10.1161/CIRCULATIONAHA.112.099044 22851544

[128] Ridker PM Everett BM Pradhan A MacFadyen JG Solomon DH Zaharris E. Low-dose methotrexate for the prevention of atherosclerotic events. N Engl J Med. (2019) 380:752–62. 10.1056/NEJMoa1809798 30415610 PMC6587584

[129] Di Francesco V Gurgone D Palomba R Ferreira MFMM Catelani T Cervadoro A. Modulating lipoprotein transcellular transport and atherosclerotic plaque formation in ApoE-/-mice via nanoformulated lipid-methotrexate conjugates. ACS Appl Mater Interfaces. (2020) 12:37943–56. 10.1021/acsami.0c12202 32805983 PMC7453397

[130] Pardoll DM. The blockade of immune checkpoints in cancer immunotherapy. Nat Rev Cancer. (2012) 12:252–64. 10.1038/nrc3239 22437870 PMC4856023

[131] Ledford H Else H Warren M. Cancer immunologists scoop medicine Nobel prize. Nature. (2018) 562:20–21. 10.1038/d41586-018-06751-0 30279600

[132] Robert C. A decade of immune-checkpoint inhibitors in cancer therapy. Nat Commun. (2020) 11:1–3. 10.1038/s41467-020-17670-y 32732879 PMC7393098

[133] Hodi FS O'Day SJ McDermott DF Weber RW Sosman JA Haanen JB. Improved survival with ipilimumab in patients with metastatic melanoma. N Engl J Med. (2010) 363:711–23. 10.1056/NEJMoa1003466 20525992 PMC3549297

[134] Twomey JD Zhang B. Cancer immunotherapy update: FDA-approved checkpoint inhibitors and companion diagnostics. AAPS J. (2021) 23:1–11. 10.1208/s12248-021-00574-0 33677681 PMC7937597

[135] Mullard A. FDA approves 100th monoclonal antibody product. Nat Rev Drug Discov. (2021) 20:491–5. 10.1038/d41573-021-00079-7 33953368

[136] Photopoulos J. The future of tissue-agnostic drugs. Nature. (2020) 585:S16–18. 10.1038/d41586-020-02679-6

[137] Haslam A Prasad V. Estimation of the percentage of US patients with cancer who are eligible for and respond to checkpoint inhibitor immunotherapy drugs. JAMA Netw Open. (2019) 2:e192535. 10.1001/jamanetworkopen.2019.2535 31050774 PMC6503493

[138] Postow MA Sidlow R Hellmann MD. Immune-related adverse events associated with immune checkpoint blockade. N Engl J Med. (2018) 378:158–68. 10.1056/NEJMra1703481 29320654

[139] Chen L Flies DB. Molecular mechanisms of T cell co-stimulation and co-inhibition. Nat Rev Immunol. (2013) 13:227–42. 10.1038/nri3405 23470321 PMC3786574

[140] Simons KH de Jong A Jukema JW de Vries MR Arens R Quax PHA. T cell co-stimulation and co-inhibition in cardiovascular disease: a double-edged sword. Nat Rev Cardiol. (2019) 16:325–43. 10.1038/s41569-019-0164-7 30770894

[141] Buchbinder EI Desai A. CTLA-4 and PD-1 pathways similarities, differences, and implications of their inhibition. Am J Clin Oncol Cancer Clin Trials. (2016) 39:98–106. 10.1097/COC.0000000000000239 26558876 PMC4892769

[142] Ramos-Casals M Brahmer JR Callahan MK Flores-Chávez A Keegan N Khamashta MA. Immune-related adverse events of checkpoint inhibitors. Nat Rev Dis Prim. (2020) 6:1–21. 10.1038/s41572-020-0160-6 32382051 PMC9728094

[143] Xu C Chen Y-P Du X-J Liu J-Q Huang C-L Chen L. Comparative safety of immune checkpoint inhibitors in cancer: systematic review and network meta-analysis. BMJ. (2018) 363:4226. 10.1136/bmj.k4226 30409774 PMC6222274

[144] Martins F Sofiya L Sykiotis GP Lamine F Maillard M Fraga M. Adverse effects of immune-checkpoint inhibitors: epidemiology, management and surveillance. Nat Rev Clin Oncol. (2019) 16:563–80. 10.1038/s41571-019-0218-0 31092901

[145] Wang DY Salem J-E Cohen J V., Chandra S, Menzer C, Ye F, et al. Fatal toxic effects associated with immune checkpoint inhibitors: a systematic review and meta-analysis. JAMA Oncol. (2018) 4:1721–8. 10.1001/jamaoncol.2018.3923 30242316 PMC6440712

[146] Dolladille C Akroun J Morice P-M Dompmartin A Ezine E Sassier M. Cardiovascular immunotoxicities associated with immune checkpoint inhibitors: a safety meta-analysis. Eur Heart J. (2021) 48:ehab618. 10.1093/eurheartj/ehab618 34529770

[147] Salem J-E Manouchehri A Moey M Lebrun-Vignes B Bastarache L Pariente A. Cardiovascular toxicities associated with immune checkpoint inhibitors: an observational, retrospective, pharmacovigilance study. Lancet Oncol. (2018) 19:1579–89. 10.1016/S1470-2045(18)30608-9 30442497 PMC6287923

[148] Poels K Neppelenbroek SIM Kersten MJ Antoni ML Lutgens E Seijkens TTP. Immune checkpoint inhibitor treatment and atherosclerotic cardiovascular disease: an emerging clinical problem. J Immunother Cancer. (2021) 9:e002916. 10.1136/jitc-2021-002916 34168005 PMC8231062

[149] Inno A Chiampan A Lanzoni L Verzè M Molon G Gori S. Immune checkpoint inhibitors and atherosclerotic vascular events in cancer patients. Front Cardiovasc Med. (2021) 8:652186. 10.3389/fcvm.2021.652186 34124192 PMC8193098

[150] Bar J Markel G Gottfried T Percik R Leibowitz-Amit R Berger R. Acute vascular events as a possibly related adverse event of immunotherapy: a single-institute retrospective study. Eur J Cancer. (2019) 120:122–31. 10.1016/j.ejca.2019.06.021 31518968

[151] Pierpont TM Limper CB Richards KL. Past, present, and future of rituximab—the world's first oncology monoclonal antibody therapy. Front Oncol. (2018) 3:163. 10.3389/fonc.2018.00163 29915719 PMC5994406

[152] Scott AM Wolchok JD Old LJ. Antibody therapy of cancer. Nat Rev Cancer. (2012) 12:278–87. 10.1038/nrc3236 22437872

[153] Zahavi D Weiner L. Monoclonal antibodies in cancer therapy. Antibodies. (2020) 9:34. 10.3390/antib9030034 32698317 PMC7551545

[154] Lee DSW Rojas OL Gommerman JL. B cell depletion therapies in autoimmune disease: advances and mechanistic insights. Nat Rev Drug Discov. (2020) 20:179–99. 10.1038/s41573-020-00092-2 33324003 PMC7737718

[155] Kyaw T Cui P Tay C Kanellakis P Hosseini H Liu E. BAFF receptor mAb treatment ameliorates development and progression of atherosclerosis in hyperlipidemic ApoE-/- mice. PLoS ONE. (2013) 8:e60430. 10.1371/journal.pone.0060430 23560095 PMC3616162

[156] Tsiantoulas D Sage AP Göderle L Ozsvar-Kozma M Murphy D Porsch F. B cell–activating factor neutralization aggravates atherosclerosis. Circulation. (2018) 138:2263–73. 10.1161/CIRCULATIONAHA.117.032790 29858401 PMC6181204

[157] Porsch F Binder CJ. Impact of B-cell-targeted therapies on cardiovascular disease. Arterioscler Thromb Vasc Biol. (2019) 39:1705–14. 10.1161/ATVBAHA.119.311996 31315439

[158] Centa M Jin H Hofste L Hellberg S Busch A Baumgartner R. Germinal center–derived antibodies promote atherosclerosis plaque size and stability. Circulation. (2019) 139:2466–82. 10.1161/CIRCULATIONAHA.118.038534 30894016

[159] Tay C Liu Y-H Kanellakis P Kallies A Li Y Cao A. Follicular B cells promote atherosclerosis via T cell–mediated differentiation into plasma cells and secreting pathogenic immunoglobulin G. Arterioscler Thromb Vasc Biol. (2018) 38:e71–84. 10.1161/ATVBAHA.117.310678 29599140

[160] Ellis LM Hicklin DJ. VEGF-targeted therapy: mechanisms of anti-tumour activity. Nat Rev Cancer. (2008) 8:579–91. 10.1038/nrc2403 18596824

[161] Arnott C Punnia-Moorthy G Tan J Sadeghipour S Bursill C Patel S. The vascular endothelial growth factor inhibitors ranibizumab and aflibercept markedly increase expression of atherosclerosis-associated inflammatory mediators on vascular endothelial cells. PLoS ONE. (2016) 11:e0150688. 10.1371/journal.pone.0150688 26959822 PMC4784900

[162] Seijkens TTP Lutgens E. Cardiovascular oncology: exploring the effects of targeted cancer therapies on atherosclerosis. Curr Opin Lipidol. (2018) 29:381–8. 10.1097/MOL.0000000000000538 30074493

[163] Mendelsohn J Baselga J. The EGF receptor family as targets for cancer therapy. Oncogene. (2000) 19:6550–65. 10.1038/sj.onc.1204082 11426640

[164] Ciardiello F Tortora G. EGFR antagonists in cancer treatment. N Engl J Med. (2009) 358:1160–74. 10.1056/NEJMra0707704 18337605

[165] Slamon DJ Clark GM Wong SG Levin WJ Ullrich A McGuire WL. Human breast cancer: correlation of relapse and survival with amplification of the HER-2/neu oncogene. Science. (1987) 235:182–91. 10.1126/science.3798106 3798106

[166] Nicholson R., Gee JM., Harper M. EGFR and cancer prognosis. Eur J Cancer. (2001) 37:9–15. 10.1016/S0959-8049(01)00231-3 11597399

[167] Slamon DJ Leyland-Jones B Shak S Fuchs H Paton V Bajamonde A. Use of chemotherapy plus a monoclonal antibody against HER2 for metastatic breast cancer that overexpresses HER2. N Engl J Med. (2001) 344:783–92. 10.1056/NEJM200103153441101 11248153

[168] Jonker DJ O'Callaghan CJ Karapetis CS Zalcberg JR Tu D Au H-J. Cetuximab for the treatment of colorectal cancer. N Engl J Med. (2009) 2:2040–8. 10.1056/NEJMoa071834 18003960

[169] Bang Y-J Cutsem E Van Feyereislova A Chung HC Shen L Sawaki A. Trastuzumab in combination with chemotherapy versus chemotherapy alone for treatment of HER2-positive advanced gastric or gastro-oesophageal junction cancer (ToGA): a phase 3, open-label, randomised controlled trial. Lancet. (2010) 376:687–97. 10.1016/S0140-6736(10)61121-X 20728210

[170] Vermorken JB Mesia R Rivera F Remenar E Kawecki A Rottey S. Platinum-based chemotherapy plus cetuximab in head and neck cancer. N Engl J Med. (2009) 359:1116–27. 10.1056/NEJMoa0802656 18784101

[171] Zamorano JL Lancellotti P Rodriguez Muñoz D Aboyans V Asteggiano R Galderisi M. 2016 ESC Position Paper on cancer treatments and cardiovascular toxicity developed under the auspices of the ESC Committee for Practice Guidelines. Eur Heart J. (2016) 37:2768–801. 10.1093/eurheartj/ehw211 27567406

[172] Gronich N Lavi I Barnett-Griness O Saliba W Abernethy DR Rennert G. Tyrosine kinase-targeting drugs-associated heart failure. Br J Cancer. (2017) 116:1366–73. 10.1038/bjc.2017.88 28399109 PMC5482733

[173] Bowles EJA Wellman R Feigelson HS Onitilo AA Freedman AN Delate T. Risk of heart failure in breast cancer patients after anthracycline and trastuzumab treatment: a retrospective cohort study. JNCI J Natl Cancer Inst. (2012) 104:1293–305. 10.1093/jnci/djs317 22949432 PMC3433392

[174] Banke A Fosbøl EL Ewertz M Videbæk L Dahl JS Poulsen MK. Long-term risk of heart failure in breast cancer patients after adjuvant chemotherapy with or without trastuzumab. JACC Hear Fail. (2019) 7:217–24. 10.1016/j.jchf.2018.09.001 30819377

[175] Crone SA Zhao Y-Y Fan L Gu Y Minamisawa S Liu Y. ErbB2 is essential in the prevention of dilated cardiomyopathy. Nat Med. (2002) 8:459–65. 10.1038/nm0502-459 11984589

[176] Dreux AC Lamb DJ Modjtahedi H Ferns GAA. The epidermal growth factor receptors and their family of ligands: their putative role in atherogenesis. Atherosclerosis. (2006) 186:38–53. 10.1016/j.atherosclerosis.2005.06.038 16076471

[177] Jian W Wei C-M Guan J-H Mo C-H Xu Y-T Zheng W-B. Association between serum HER2/ErbB2 levels and coronary artery disease: a case–control study. J Transl Med. (2020) 18:1–10. 10.1186/s12967-020-02292-1 32160892 PMC7066824

[178] Mindur JE Swirski FK. Growth factors as immunotherapeutic targets in cardiovascular disease. Arterioscler Thromb Vasc Biol. (2019) 39:1275–87. 10.1161/ATVBAHA.119.311994 31092009 PMC6613384

[179] Hoyer FF Naxerova K Schloss MJ Hulsmans M Nair A V., Dutta P, et al. Tissue-specific macrophage responses to remote injury impact the outcome of subsequent local immune challenge. Immunity. (2019) 51:899–914.e7. 10.1016/j.immuni.2019.10.010 31732166 PMC6892583

[180] Berraondo P Sanmamed MF Ochoa MC Etxeberria I Aznar MA Pérez-Gracia JL. Cytokines in clinical cancer immunotherapy. Br J Cancer. (2018) 120:6–15. 10.1038/s41416-018-0328-y 30413827 PMC6325155

[181] Conlon KC Miljkovic MD Waldmann TA. Cytokines in the treatment of cancer. J Interf Cytokine Res. (2019) 39:6. 10.1089/jir.2018.0019 29889594 PMC6350412

[182] Chen H-J Tas SW de Winther MPJ. Type-I interferons in atherosclerosis. J Exp Med. (2020) 217:e20190459. 10.1084/jem.20190459 31821440 PMC7037237

[183] Boshuizen MCS de Winther MPJ. Interferons as essential modulators of atherosclerosis. Arterioscler Thromb Vasc Biol. (2015) 35:1579–88. 10.1161/ATVBAHA.115.305464 25953648

[184] Li J Fu Q Cui H Qu B Pan W Shen N. Interferon-α priming promotes lipid uptake and macrophage-derived foam cell formation: a novel link between interferon-α and atherosclerosis in lupus. Arthritis Rheum. (2011) 63:492–502. 10.1002/art.30165 21280004

[185] Lai J-H Hung L-F Huang C-Y Wu D-W Wu C-H Ho L-J. Mitochondrial protein CMPK2 regulates IFN alpha-enhanced foam cell formation, potentially contributing to premature atherosclerosis in SLE. Arthritis Res Ther. (2021) 23:1–12. 10.1186/s13075-021-02470-6 33874983 PMC8054390

[186] Srivastava S Koch MA Pepper M Campbell DJ. Type I interferons directly inhibit regulatory T cells to allow optimal antiviral T cell responses during acute LCMV infection. J Exp Med. (2014) 211:961–74. 10.1084/jem.20131556 24711580 PMC4010906

[187] Gangaplara A Martens C Dahlstrom E Metidji A Gokhale AS Glass DD. Type I interferon signaling attenuates regulatory T cell function in viral infection and in the tumor microenvironment. PLoS Pathog. (2018) 14:e1006985. 10.1371/journal.ppat.1006985 29672594 PMC5929570

[188] Niessner A Sato K Chaikof EL Colmegna I Goronzy JJ Weyand CM. Pathogen-sensing plasmacytoid dendritic cells stimulate cytotoxic T-cell function in the atherosclerotic plaque through interferon-α. Circulation. (2006) 114:2482–9. 10.1161/CIRCULATIONAHA.106.642801 17116765

[189] Niessner A Shin MS Pryshchep O Goronzy JJ Chaikof EL Weyand CM. Synergistic proinflammatory effects of the antiviral cytokine interferon-α and toll-like receptor 4 ligands in the atherosclerotic plaque. Circulation. (2007) 116:2043–52. 10.1161/CIRCULATIONAHA.107.697789 17938289

[190] Denny MF Thacker S Mehta H Somers EC Dodick T Barrat FJ. Interferon-α promotes abnormal vasculogenesis in lupus: a potential pathway for premature atherosclerosis. Blood. (2007) 110:2907–15. 10.1182/blood-2007-05-089086 17638846 PMC2018671

[191] Thacker SG Berthier CC Mattinzoli D Rastaldi MP Kretzler M Kaplan MJ. The detrimental effects of IFN-α on vasculogenesis in lupus are mediated by repression of IL-1 pathways: potential role in atherogenesis and renal vascular rarefaction. J Immunol. (2010) 185:4457–69. 10.4049/jimmunol.1001782 20805419 PMC2978924

[192] Buie JJ Renaud LL Muise-Helmericks R Oates JC. IFN-α negatively regulates the expression of endothelial nitric oxide synthase and nitric oxide production: implications for systemic lupus erythematosus. J Immunol. (2017) 199:1979–88. 10.4049/jimmunol.1600108 28779021 PMC5587385

[193] Esdaile JM Abrahamowicz M Grodzicky T Li Y Panaritis C Du Berger R. Traditional framingham risk factors fail to fully account for accelerated atherosclerosis in systemic lupus erythematosus. Arthritis Rheum. (2001) 44:2331–7. 10.1002/1529-0131(200110)44:10<2331::AID-ART395>3.0.CO;2-I 11665973

[194] Malek TR. The biology of interleukin-2. Annu Rev Immunol. (2008) 26:453–79. 10.1146/annurev.immunol.26.021607.090357 18062768

[195] Malek TR Bayer AL. Tolerance, not immunity, crucially depends on IL-2. Nat Rev Immunol. (2004) 4:665–74. 10.1038/nri1435 15343366

[196] Rosenberg SA. IL-2: the first effective immunotherapy for human cancer. J Immunol. (2014) 192:5451–8. 10.4049/jimmunol.1490019 24907378 PMC6293462

[197] Sadlack B Merz H Schorle H Schimpl A Feller AC Horak I. Ulcerative colitis-like disease in mice with a disrupted interleukin-2 gene. Cell. (1993) 75:253–61. 10.1016/0092-8674(93)80067-O 8402910

[198] Sadlack B Löhler J Schorle H Klebb G Haber H Sickel E. Generalized autoimmune disease in interleukin-2-deficient mice is triggered by an uncontrolled activation and proliferation of CD4+ T cells. Eur J Immunol. (1995) 25:3053–9. 10.1002/eji.1830251111 7489743

[199] Abbas AK Trotta E Simeonov DR Marson A Bluestone JA. Revisiting IL-2: biology and therapeutic prospects. Sci Immunol. (2018) 3:eaat1482. 10.1126/sciimmunol.aat1482 29980618

[200] Koreth J Matsuoka KI Kim HT McDonough SM Bindra B Alyea EP. Interleukin-2 and regulatory T cells in graft-versus-host disease. N Engl J Med. (2011) 365:2055–66. 10.1056/NEJMoa1108188 22129252 PMC3727432

[201] Matsuoka KI Koreth J Kim HT Bascug G McDonough S Kawano Y. Low-dose interleukin-2 therapy restores regulatory T cell homeostasis in patients with chronic graft-versus-host disease. Sci Transl Med. (2013) 5:179ra43. 10.1126/scitranslmed.3005265 23552371 PMC3686517

[202] Elkind MSV Rundek T Sciacca RR Ramas R Chen HJ Boden-Albala B. Interleukin-2 levels are associated with carotid artery intima-media thickness. Atherosclerosis. (2005) 180:181–7. 10.1016/j.atherosclerosis.2004.11.015 15823291

[203] Ding R Gao W Ostrodci DH He Z Song Y Ma L. Effect of interleukin-2 level and genetic variants on coronary artery disease. Inflammation. (2013) 36:1225–31. 10.1007/s10753-013-9659-2 23715819

[204] Foks AC Frodermann V ter Borg M Habets KLL Bot I Zhao Y. Differential effects of regulatory T cells on the initiation and regression of atherosclerosis. Atherosclerosis. (2011) 218:53–60. 10.1016/j.atherosclerosis.2011.04.029 21621777

[205] Tomala J Chmelova H Mrkvan T Rihova B Kovar M. In vivo expansion of activated naive CD8+ T cells and NK cells driven by complexes of IL-2 and anti-IL-2 monoclonal antibody as novel approach of cancer immunotherapy. J Immunol. (2009) 183:4904–12. 10.4049/jimmunol.0900284 19801515

[206] Spangler JB Tomala J Luca VC Jude KM Dong S Ring AM. Antibodies to interleukin-2 elicit selective T cell subset potentiation through distinct conformational mechanisms. Immunity. (2015) 42:815–25. 10.1016/j.immuni.2015.04.015 25992858 PMC4439582

[207] Trotta E Bessette PH Silveria SL Ely LK Jude KM Le DT. A human anti-IL-2 antibody that potentiates regulatory T cells by a structure-based mechanism. Nat Med. (2018) 24:1005–14. 10.1038/s41591-018-0070-2 29942088 PMC6398608

[208] Zhao TX Kostapanos M Griffiths C Arbon EL Hubsch A Kaloyirou F. Low-dose interleukin-2 in patients with stable ischaemic heart disease and acute coronary syndromes (LILACS): protocol and study rationale for a randomised, double-blind, placebo-controlled, phase I/II clinical trial. BMJ Open. (2018) 8:e022452. 10.1136/bmjopen-2018-022452 30224390 PMC6144322

[209] Kozmiński P Halik PK Chesori R Gniazdowska E. Overview of dual-acting drug methotrexate in different neurological diseases, autoimmune pathologies and cancers. Int J Mol Sci. (2020) 21:3483. 10.3390/ijms21103483 32423175 PMC7279024

[210] Farber S Diamond LK Mercer RD Sylvester RFJ Wolff JA. Temporary remissions in acute leukemia in children produced by folic acid antagonist, 4-aminopteroyl-glutamic acid (Aminopterin). N Engl J Med. (1948) 238:787–93. 10.1056/NEJM194806032382301 18860765

[211] Goldin A Venditti JM Humphreys SR Dennis D Mantel N Greenhouse SW. a quantitative comparison of the antileukemic effectiveness of two folic acid antagonists in mice. JNCI J Natl Cancer Inst. (1955) 15:1657–64. 14381889

[212] WHO. WHO Model List of Essential Medicines - 22nd List, 2021. (2021). Available online at: https://www.who.int/publications/i/item/WHO-MHP-HPS-EML-2021.02 (accessed December 16, 2021).

[213] Cronstein BN Aune TM. Methotrexate and its mechanisms of action in inflammatory arthritis. Nat Rev Rheumatol. (2020) 16:145–54. 10.1038/s41584-020-0373-9 32066940

[214] Weinblatt ME Coblyn JS Fox DA Fraser PA Holdsworth DE Glass DN. Efficacy of low-dose methotrexate in rheumatoid arthritis. N Engl J Med. (2010) 16:287–95. 10.1056/NEJM198503283121303 3883172

[215] Giannini EH Brewer EJ Kuzmina N Shaikov A Maximov A Vorontsov I. Methotrexate in resistant juvenile rheumatoid arthritis. N Engl J Med. (2010) 326:1043–9. 10.1056/NEJM199204163261602 1549149

[216] Choi HK Hernán MA Seeger JD Robins JM Wolfe F. Methotrexate and mortality in patients with rheumatoid arthritis: a prospective study. Lancet. (2002) 359:1173–7. 10.1016/S0140-6736(02)08213-2 11955534

[217] Micha R Imamura F Wyler Von Ballmoos M Solomon DH Hernán MA Ridker PM. Systematic review and meta-analysis of methotrexate use and risk of cardiovascular disease. Am J Cardiol. (2011) 108:1362–70. 10.1016/j.amjcard.2011.06.054 21855836 PMC3196048

[218] Lutgens E Atzler D Döring Y Duchene J Steffens S Weber C. Immunotherapy for cardiovascular disease. Eur Heart J. (2019) 40:3937–46. 10.1093/eurheartj/ehz283 31121017

[219] Tardif J-C Kouz S Waters DD Bertrand OF Diaz R Maggioni AP. Efficacy and safety of low-dose colchicine after myocardial infarction. N Engl J Med. (2019) 381:2497–505. 10.1056/NEJMoa1912388 31733140

[220] Nidorf SM Fiolet ATL Mosterd A Eikelboom JW Schut A Opstal TSJ. Colchicine in patients with chronic coronary disease. N Engl J Med. (2020) 383:1838–47. 10.1056/NEJMoa2021372 32865380

[221] Imazio M Andreis A Brucato A Adler Y De Ferrari GM. Colchicine for acute and chronic coronary syndromes. Heart. (2020) 106:1555–60. 10.1136/heartjnl-2020-317108 32611559

[222] Raal FJ Kallend D Ray KK Turner T Koenig W Wright RS. Inclisiran for the treatment of heterozygous familial hypercholesterolemia. N Engl J Med. (2020) 382:1520–30. 10.1056/NEJMoa1913805 32197277

[223] Ray KK Wright RS Kallend D Koenig W Leiter LA Raal FJ. Two phase 3 trials of inclisiran in patients with elevated LDL cholesterol. N Engl J Med. (2020) 382:1507–19. 10.1056/NEJMoa1912387 32187462

[224] Ridker PM MacFadyen JG Thuren T Everett B Libby P Glynn R. Effect of interleukin-1β inhibition with canakinumab on incident lung cancer in patients with atherosclerosis: exploratory results from a randomised, double-blind, placebo-controlled trial. Lancet. (2017) 390:1833–42. 10.1016/S0140-6736(17)32247-X 28855077

[225] Voronov E Shouval DS Krelin Y Cagnano E Benharroch D Iwakura Y. IL-1 is required for tumor invasiveness and angiogenesis. Proc Natl Acad Sci. (2003) 100:2645–50. 10.1073/pnas.0437939100 12598651 PMC151394

[226] Liu X Bao X Hu M Chang H Jiao M Cheng J. Inhibition of PCSK9 potentiates immune checkpoint therapy for cancer. Nature. (2020) 588:693–8. 10.1038/s41586-020-2911-7 33177715 PMC7770056

[227] Quang CT Zaniboni B Humeau R Lengliné E Dourthe ME Ganesan R. Preclinical efficacy of humanized, non–FcγR-binding anti-CD3 antibodies in T-cell acute lymphoblastic leukemia. Blood. (2020) 136:1298–302. 10.1182/blood.2019003801 32483610

[228] Kuo MC Chang SJ Hsieh MC. Colchicine significantly reduces incident cancer in gout male patients: a 12-year cohort study. Medicine. (2015) 94:e1570. 10.1097/MD.0000000000001570 26683907 PMC5058879

[229] Fakih M Replogle T Lehr JE Pienta KJ Yagoda A. Inhibition of prostate cancer growth by estramustine and colchicine. Prostate. (1995) 26:310–15. 10.1002/pros.2990260606 7784270

[230] Cho JH Joo YH Shin EY Park EJ Kim SM. Anticancer effects of colchicine on hypopharyngeal cancer. Anticancer Res. (2017) 37:6269 LP-80. 10.21873/anticanres.12078 29061810

[231] Lin ZY Wu CC Chuang YH Chuang WL. Anti-cancer mechanisms of clinically acceptable colchicine concentrations on hepatocellular carcinoma. Life Sci. (2013) 93:323–8. 10.1016/j.lfs.2013.07.002 23871804

[232] Craig DH Owen CR Conway WC Walsh MF Downey C Basson MD. Colchicine inhibits pressure-induced tumor cell implantation within surgical wounds and enhances tumor-free survival in mice. J Clin Invest. (2008) 118:3170–80. 10.1172/JCI34279 18704196 PMC2515382

[233] Bent R Moll L Grabbe S Bros M. Interleukin-1 beta—a friend or foe in malignancies? Int J Mol Sci. (2018) 19:2155. 10.3390/ijms19082155 30042333 PMC6121377

[234] Greten FR Grivennikov SI. Inflammation and cancer: triggers, mechanisms, and consequences. Immunity. (2019) 51:27–41. 10.1016/j.immuni.2019.06.025 31315034 PMC6831096

[235] Barlesi F Cho BC Castro G Marchi PD Felip E Goto Y. The CANOPY program: Canakinumab in patients (pts) with non-small cell lung cancer (NSCLC). J Clin Oncol. (2019) 37:TPS9124. 10.1200/JCO.2019.37.15_SUPPL.TPS9124

[236] Garrido P Pujol J-L Kim ES Lee JM Tsuboi M Gómez-Rueda A. Canakinumab with and without pembrolizumab in patients with resectable non-small-cell lung cancer: CANOPY-N study design. Fut Oncol. (2021) 17:1459–72. 10.2217/fon-2020-1098 33648347

[237] Novartis Provides Update on Phase III Study Evaluating Canakinumab (ACZ885) as Second or Third-Line Treatment in Combination With Chemotherapy in Non-Small Cell Lung Cancer. Novartis. Available online at: https://www.novartis.com/news/media-releases/novartis-provides-update-phase-iii-study-evaluating-canakinumab-acz885-second-or-third-line-treatment-combination-chemotherapy-non-small-cell-lung-cancer (accessed October 13, 2021).

[238] Dasgeb B Kornreich D McGuinn K Okon L Brownell I Sackett DL. Colchicine: an ancient drug with novel applications. Br J Dermatol. (2018) 178:350–6. 10.1111/bjd.15896 28832953 PMC5812812

[239] FitzGerald JD Dalbeth N Mikuls T Brignardello-Petersen R Guyatt G Abeles AM. 2020 American College of Rheumatology guideline for the management of gout. Arthritis Care Res. (2020) 72:744–60. 10.1002/acr.24180 32391934 PMC10563586

[240] Tong DC Quinn S Nasis A Hiew C Roberts-Thomson P Adams H. Colchicine in patients with acute coronary syndrome. Circulation. (2020) 142:1890–900. 10.1161/CIRCULATIONAHA.120.050771 32862667

[241] Fiolet ATL Collaboration the CCT Opstal TSJ Collaboration the CCT Mosterd A Collaboration the CCT . Efficacy and safety of low-dose colchicine in patients with coronary disease: a systematic review and meta-analysis of randomized trials. Eur Heart J. (2021) 42:2765–75. 10.1093/eurheartj/ehab115 33769515

[242] Kofler T Kurmann R Lehnick D Cioffi GM Chandran S Attinger-Toller A. Colchicine in patients with coronary artery disease: a systematic review and meta-analysis of randomized trials. J Am Heart Assoc. (2021) 10:21198. 10.1161/JAHA.121.021198 34369166 PMC8475038

[243] Kumar A Sharma PR Mondhe DM. Potential anticancer role of colchicine-based derivatives: an overview. Anticancer Drugs. (2016) 28:250–62. 10.1097/CAD.0000000000000464 28030380

[244] Wen C-C Chen H-M Chen S-S Huang L-T Chang W-T Wei W-C. Specific microtubule-depolymerizing agents augment efficacy of dendritic cell-based cancer vaccines. J Biomed Sci. (2011) 18:1–15. 10.1186/1423-0127-18-44 21689407 PMC3141632

[245] Hess CN Wang CCL Hiatt WR. PCSK9 Inhibitors: mechanisms of action, metabolic effects, and clinical outcomes. Annu Rev Med. (2018) 69:133–45. 10.1146/annurev-med-042716-091351 29095667

[246] Mach F Baigent C Catapano AL Koskinas KC Casula M Badimon L. 2019 ESC/EAS Guidelines for the management of dyslipidaemias: lipid modification to reduce cardiovascular risk. Eur Heart J. (2020) 41:111–88. 10.15829/1560-4071-2020-3826 31504418

[247] Kuzu OF Noory MA Robertson GP. The role of cholesterol in cancer. Cancer Res. (2016) 76:2063–70. 10.1158/0008-5472.CAN-15-2613 27197250 PMC5813477

[248] Shi M Zheng H Nie B Gong W Cui X. Statin use and risk of liver cancer: an update meta-analysis. BMJ Open. (2014) 4:e005399. 10.1136/bmjopen-2014-005399 25227628 PMC4166249

[249] Pradelli D Soranna D Zambon A Catapano A Mancia G Vecchia C La. Statins use and the risk of all and subtype hematological malignancies: a meta-analysis of observational studies. Cancer Med. (2015) 4:770–80. 10.1002/cam4.411 25809667 PMC4430269

[250] Ying Y Ling Y Yang L Huang H Hu X Zhao C. Prognostic significance of statin use in colorectal cancer. Medicine. (2015) 94:e908. 10.1097/MD.0000000000000908 26107680 PMC4504590

[251] Dale KM Coleman CI Henyan NN Kluger J White CM. Statins and cancer risk: a meta-analysis. JAMA. (2006) 295:74–80. 10.1001/jama.295.1.74 16391219

[252] Tan P Wei S Tang Z Gao L Zhang C Nie P. LDL-lowering therapy and the risk of prostate cancer: a meta-analysis of 6 randomized controlled trials and 36 observational studies. Sci Rep. (2016) 6:1–9. 10.1038/srep24521 27075437 PMC4830970

[253] Momtazi-Borojeni AA Jaafari MR Badiee A Sahebkar A. Long-term generation of antiPCSK9 antibody using a nanoliposome-based vaccine delivery system. Atherosclerosis. (2019) 283:69–78. 10.1016/j.atherosclerosis.2019.02.001 30797988

[254] Kuhn C Weiner HL. Therapeutic anti-CD3 monoclonal antibodies: from bench to bedside. Immunotherapy. (2016) 8:889–906. 10.2217/imt-2016-0049 27161438

[255] Belghith M Bluestone JA Barriot S Mégret J Bach JF Chatenoud L. TGF-β-dependent mechanisms mediate restoration of self-tolerance induced by antibodies to CD3 in overt autoimmune diabetes. Nat Med. (2003) 9:1202–8. 10.1038/nm924 12937416

[256] Perruche S Zhang P Liu Y Saas P Bluestone JA Chen WJ. CD3-specific antibody–induced immune tolerance involves transforming growth factor-β from phagocytes digesting apoptotic T cells. Nat Med. (2008) 14:528–35. 10.1038/nm1749 18438416

[257] Weinshenker BG Bass B Karlik S Ebers GC Rice GPA. An open trial of OKT3 in patients with multiple sclerosis. Neurology. (1991) 41:1047. 10.1212/WNL.41.7.1047 1906145

[258] Keymeulen B Vandemeulebroucke E Ziegler AG Mathieu C Kaufman L Hale G. Insulin needs after CD3-antibody therapy in new-onset type 1 diabetes. N Engl J Med. (2005) 352:2598–608. 10.1056/NEJMoa043980 15972866

[259] Hagopian W Ferry RJ Sherry N Carlin D Bonvini E Johnson S. Teplizumab preserves C-peptide in recent-onset type 1 diabetes: Two-year results from the randomized, placebo-controlled Protégé trial. Diabetes. (2013) 62:3901–8. 10.2337/db13-0236 23801579 PMC3806608

[260] Herold KC Gitelman SE Ehlers MR Gottlieb PA Greenbaum CJ Hagopian W. Teplizumab (Anti-CD3 mAb) treatment preserves C-peptide responses in patients with new-onset type 1 diabetes in a randomized controlled trial: metabolic and immunologic features at baseline identify a subgroup of responders. Diabetes. (2013) 62:3766–74. 10.2337/db13-0345 23835333 PMC3806618

[261] Herold KC Bundy BN Long SA Bluestone JA DiMeglio LA Dufort MJ. An anti-CD3 antibody, teplizumab, in relatives at risk for type 1 diabetes. N Engl J Med. (2019) 381:603–13. 10.1056/NEJMoa1902226 31180194 PMC6776880

[262] Plevy S Salzberg B Van Assche G Regueiro M Hommes D Sandborn W. A phase I study of visilizumab, a humanized anti-CD3 monoclonal antibody, in severe steroid-refractory ulcerative colitis. Gastroenterology. (2007) 133:1414–22. 10.1053/j.gastro.2007.08.035 17920064

[263] Kita T Yamashita T Sasaki N Kasahara K Sasaki Y Yodoi K. Regression of atherosclerosis with anti-CD3 antibody via augmenting a regulatory T-cell response in mice. Cardiovasc Res. (2014) 102:107–17. 10.1093/cvr/cvu002 24403315

[264] Sasaki N Yamashita T Takeda M Shinohara M Nakajima K Tawa H. Oral anti-CD3 antibody treatment induces regulatory t cells and inhibits the development of atherosclerosis in mice. Circulation. (2009) 120:1996–2005. 10.1161/CIRCULATIONAHA.109.863431 19884470

[265] Steffens S Burger F Pelli G Dean Y Elson G Kosco-Vilbois M. Short-term treatment with anti-CD3 antibody reduces the development and progression of atherosclerosis in mice. Circulation. (2006) 114:1977–84. 10.1161/CIRCULATIONAHA.106.627430 17043169

[266] Gramatzki M Burger R Strobel G Trautmann U Bartram CR Helm G. Therapy with OKT3 monoclonal antibody in refractory T cell acute lymphoblastic leukemia induces interleukin-2 responsiveness. Leukemia. (1995) 9:382–90. 7885036

[267] Sosman JA Kefer C Fisher RI Jacobs CD Pumfery P Ellis TM. A phase IA/IB trial of anti-CD3 murine monoclonal antibody plus low-dose continuous-infusion interleukin-2 in advanced cancer patients. J Immunother. (1995) 17:171–80. 10.1097/00002371-199504000-00006 7613643

[268] Sosman JA Weiss GR Margolin KA Aronson FR Sznol M Atkins MB. Phase IB clinical trial of anti-CD3 followed by high-dose bolus interleukin-2 in patients with metastatic melanoma and advanced renal cell carcinoma: clinical and immunologic effects. J Clin Oncol. (1993) 11:1496–505. 10.1200/JCO.1993.11.8.1496 8336188

[269] Feins S Kong W Williams EF Milone MC Fraietta JA. An introduction to chimeric antigen receptor (CAR) T-cell immunotherapy for human cancer. Am J Hematol. (2019) 94:S3–9. 10.1002/ajh.25418 30680780

[270] Schuster SJ Svoboda J Chong EA Nasta SD Mato AR Anak Ö. Chimeric antigen receptor T cells in refractory B-cell lymphomas. N Engl J Med. (2017) 377:2545–54. 10.1056/NEJMoa1708566 29226764 PMC5788566

[271] Neelapu SS Locke FL Bartlett NL Lekakis LJ Miklos DB Jacobson CA. Axicabtagene ciloleucel CAR T-cell therapy in refractory large B-cell lymphoma. N Engl J Med. (2017) 377:2531–44. 10.1056/NEJMoa1707447 29226797 PMC5882485

[272] Wang M Munoz J Goy A Locke FL Jacobson CA Hill BT. KTE-X19 CAR T-cell therapy in relapsed or refractory mantle-cell lymphoma. N Engl J Med. (2020) 382:1331–42. 10.1056/NEJMoa1914347 32242358 PMC7731441

[273] Abramson JS Palomba ML Gordon LI Lunning MA Wang M Arnason J. Lisocabtagene maraleucel for patients with relapsed or refractory large B-cell lymphomas (TRANSCEND NHL 001): a multicentre seamless design study. Lancet. (2020) 396:839–52. 10.1016/S0140-6736(20)31366-0 32888407

[274] Munshi NC Anderson LD Shah N Madduri D Berdeja J Lonial S. Idecabtagene vicleucel in relapsed and refractory multiple myeloma. N Engl J Med. (2021) 384:705–16. 10.1056/NEJMoa2024850 33626253

[275] Neelapu SS Tummala S Kebriaei P Wierda W Gutierrez C Locke FL., Lin Y, Jain N, Daver N, et al. Chimeric antigen receptor T-cell therapy — assessment and management of toxicities. Nat Rev Clin Oncol. (2017) 15:47–62. 10.1038/nrclinonc.2017.148 28925994 PMC6733403

[276] Alvi RM Frigault MJ Fradley MG Jain MD Mahmood SS Awadalla M. Cardiovascular events among adults treated with chimeric antigen receptor T-cells (CAR-T). J Am Coll Cardiol. (2019) 74:3099–108. 10.1016/j.jacc.2019.10.038 31856966 PMC6938409

[277] Bayati F Mohammadi M Valadi M Jamshidi S Foma AM Sharif-Paghaleh E. The therapeutic potential of regulatory T cells: challenges and opportunities. Front Immunol. (2021) 11:3455. 10.3389/fimmu.2020.585819 33519807 PMC7844143

[278] Marek-Trzonkowska N Myśliwiec M Dobyszuk A Grabowska M Derkowska I Juścińska J. Therapy of type 1 diabetes with CD4+CD25highCD127-regulatory T cells prolongs survival of pancreatic islets — results of one year follow-up. Clin Immunol. (2014) 153:23–30. 10.1016/j.clim.2014.03.016 24704576

[279] Bluestone JA Buckner JH Fitch M Gitelman SE Gupta S Hellerstein MK. Type 1 diabetes immunotherapy using polyclonal regulatory T cells. Sci Transl Med. (2015) 7:315ra189. 10.1126/scitranslmed.aad4134 26606968 PMC4729454

[280] Brunstein CG Miller JS Cao Q McKenna DH Hippen KL Curtsinger J. Infusion of *ex vivo* expanded T regulatory cells in adults transplanted with umbilical cord blood: safety profile and detection kinetics. Blood. (2011) 117:1061–70. 10.1182/blood-2010-07-293795 20952687 PMC3035067

[281] Brunstein CG Miller JS McKenna DH Hippen KL DeFor TE Sumstad D. Umbilical cord blood–derived T regulatory cells to prevent GVHD: kinetics, toxicity profile, and clinical effect. Blood. (2016) 127:1044–51. 10.1182/blood-2015-06-653667 26563133 PMC4768428

[282] Mathew JM H-Voss J LeFever A Konieczna I Stratton C He J. A phase I clinical trial with *ex vivo* expanded recipient regulatory T cells in living donor kidney transplants. Sci Rep. (2018) 8:1–12. 10.1038/s41598-018-25574-7 29743501 PMC5943280

[283] Desreumaux P Foussat A Allez M Beaugerie L Hébuterne X Bouhnik Y. Safety and efficacy of antigen-specific regulatory T-cell therapy for patients with refractory Crohn's disease. Gastroenterology. (2012) 143:1207–17.e2. 10.1053/j.gastro.2012.07.116 22885333

[284] Ait-Oufella H Salomon BL Potteaux S Robertson A-KL Gourdy P Zoll J. Natural regulatory T cells control the development of atherosclerosis in mice. Nat Med. (2006) 12:178–80. 10.1038/nm1343 16462800

[285] Yang K Li D Luo M Hu Y. Generation of HSP60-specific regulatory T cell and effect on atherosclerosis. Cell Immunol. (2006) 243:90–95. 10.1016/j.cellimm.2007.01.002 17324390

[286] Palinski W Miller E Witztum JL. Immunization of low density lipoprotein (LDL) receptor-deficient rabbits with homologous malondialdehyde-modified LDL reduces atherogenesis. Proc Natl Acad Sci USA. (1995) 92:821–5. 10.1073/pnas.92.3.821 7846059 PMC42712

[287] Klingenberg R Lebens M Hermansson A Fredrikson GN Strodthoff D Rudling M. Intranasal immunization with an apolipoprotein B-100 fusion protein induces antigen-specific regulatory T cells and reduces atherosclerosis. Arterioscler Thromb Vasc Biol. (2010) 30:946–52. 10.1161/ATVBAHA.109.202671 20167655

[288] Herbin O Ait-Oufella H Yu W Fredrikson GN Aubier B Perez N. Regulatory T-cell response to apolipoprotein B100-derived peptides reduces the development and progression of atherosclerosis in mice. Arterioscler Thromb Vasc Biol. (2012) 32:605–12. 10.1161/ATVBAHA.111.242800 22223728

[289] Gisterå A Klement ML Polyzos KA Mailer RKW Duhlin A Karlsson MCI. Low-density lipoprotein-reactive T cells regulate plasma cholesterol levels and development of atherosclerosis in humanized hypercholesterolemic mice. Circulation. (2018) 138:2513–26. 10.1161/CIRCULATIONAHA.118.034076 29997115 PMC6254780

[290] Krienke C Kolb L Diken E Streuber M Kirchhoff S Bukur T. A noninflammatory mRNA vaccine for treatment of experimental autoimmune encephalomyelitis. Science. (2021) 371:145–53. 10.1126/science.aay3638 33414215

[291] Saxena M van der Burg SH Melief CJM Bhardwaj N. Therapeutic cancer vaccines. Nat Rev Cancer. (2021) 21:360–78. 10.1038/s41568-021-00346-0 33907315

[292] Sahin U Türeci Ö. Personalized vaccines for cancer immunotherapy. Science. (2018) 359:1355–60. 10.1126/science.aar7112 29567706

[293] Carreno BM Magrini V Becker-Hapak M Kaabinejadian S Hundal J Petti AA. A dendritic cell vaccine increases the breadth and diversity of melanoma neoantigen-specific T cells. Science. (2015) 348:803–8. 10.1126/science.aaa3828 25837513 PMC4549796

